# Effect of exercise and/or educational interventions on physical activity and pain in patients with hip/knee osteoarthritis: A systematic review with meta-analysis

**DOI:** 10.1371/journal.pone.0275591

**Published:** 2022-11-21

**Authors:** Ryo Sasaki, Yuichiro Honda, Satoshi Oga, Takuya Fukushima, Natsumi Tanaka, Yasuhiro Kajiwara, Koichi Nakagawa, Ayumi Takahashi, Yukinori Sakamoto, Hinako Morita, Yutaro Kondo, Seima Okita, Yasutaka Kondo, Kyo Goto, Hideki Kataoka, Junya Sakamoto, Minoru Okita

**Affiliations:** 1 Department of Physical Therapy Science, Nagasaki University Graduate School of Biomedical Sciences, Nagasaki, Japan; 2 Department of Rehabilitation, Juzenkai Hospital, Nagasaki, Japan; 3 Institute of Biomedical Sciences (Health Sciences), Nagasaki University, Nagasaki, Japan; 4 Department of Physical Therapy, Faculty of Rehabilitation, Kobe Gakuin University, Hyogo, Japan; 5 Department of Physical Medicine and Rehabilitation, Kansai Medical University, Osaka, Japan; 6 Department of Physical Therapy, School of Rehabilitation Sciences, Seirei Christopher University, Shizuoka, Japan; 7 Department of Rehabilitation, Nagasaki University Hospital, Nagasaki, Japan; 8 Department of Rehabilitation, Nagasaki Memorial Hospital, Nagasaki, Japan; 9 Department of Rehabilitation, Japanese Red Cross Nagasaki Genbaku Hospital, Nagasaki, Japan; Sport Sciences School of Rio Maior - Politechnic Institute of Santarem, PORTUGAL

## Abstract

**Objective:**

To investigate the effectiveness of exercise and/or educational intervention on physical activity and pain in patients with hip/knee osteoarthritis (OA) using systematic review and meta-analysis.

**Methods:**

We searched randomized controlled trials that investigated physical activity and pain and compared exercise and/or educational intervention with usual care in patients with hip/knee OA in MEDLINE (PubMed), ProQuest, Scopus, and the Physiotherapy Evidence Database (PEDro), including all those published by April 30, 2022 and written in English. Studies that newly applied analgesics after onset of the intervention were excluded. The revised Cochrane risk-of-bias tool for randomized trials was used to assess the methodological qualities. The random-effects model was used for meta-analysis with standard mean differences using RevMan version 5.4. The body of evidence for each study was synthesized using the Grading of Recommendations, Assessment, Development, and Evaluation (GRADE) approach.

**Results:**

Twenty studies including 2,350 patients were included (7 exercise studies, 8 educational intervention studies and 5 combination studies). The meta-analysis demonstrated that there is very low evidence that combination therapy of exercise and educational intervention improve the physical activity level at the endpoint (4 articles; SMD 0.33, 95% CI 0.04 to 0.51, P = 0.03). Low evidence was observed for combination therapy reducing pain (4 articles; SMD -0.15, 95% CI -0.29 to -0.02, P = 0.03).

**Discussion:**

The current evidence indicated that combination therapy of exercise and educational intervention leads to improved physical activity and pain reduction in hip/knee OA patients, but the risk of bias in each study, especially in allocation concealment, downgraded the evidence level. These findings support the use of a combination therapy of exercise and educational intervention to promote physical activity levels in patients with hip/knee OA.

**Trail registration:**

There was no financial support for this research. The protocol was registered at the International Prospective Register of Systematic Reviews (registration code: CRD42020205804).

## Introduction

Osteoarthritis (OA) is a locomotive disease that has a high incidence in the lower limbs, particularly in the hip and knee joints. It is estimated that 24% of the general adult population suffers from osteoarthritis [1]. The prevalence of hip OA is reported to be 27% in individuals over the age of 45 years, and that of knee OA is 37.4% in individuals over 60 years [2], and up to 80% in those over 65 years in high-income countries [1].

A low level of physical activity in patients with hip or knee OA undergoing non-surgical treatment is associated with greater disease severity [3–7] and is known to worsen pain [8–10] and physical function [9, 11, 12] in OA patients. Pain and loss of physical function lead to more time spent in sedentary behavior. This dynamic contributes to a vicious circle in which more sedentary time leads to increased pain and loss of physical function [12, 13]. Conversely, higher levels of physical activity reduce disease severity and improve muscle strength and gait function in patients with hip or knee OA [4, 7, 9, 11, 12, 14]. Therefore, management to improve physical activity is important in patients with hip or knee OA.

Exercise therapy and/or educational intervention is recommended as the first-line treatment for hip or knee OA [15–19]. However, previous meta-analyses have reported that high-intensity exercise and educational intervention did not improve long-term physical activity in patients with hip or knee OA [20, 21]. These results may have been influenced by the condition of the group setting and differences in the time points of each outcome measure. Specifically, in the group setting, since the control group included various interventions, such as exercise, educational intervention, and physical therapy, the effect on physical activity could not be accurately compared with the intervention group. Additionally, the overlap of the endpoints and follow-up examinations made it impossible to examine the factor of the timing of the evaluation. Therefore, it is necessary to establish these conditions in detail and to re-examine the effects of exercise or educational intervention on increasing long-term physical activity in patients with OA. Moreover, in recent years, the combination therapy of exercise and educational intervention has been attracting attention, and the effects of combination therapy on physical activity have been reported at the randomized controlled trial (RCT) level. Previous RCTs have reported that such combination therapy improved long-term physical activity in patients with hip and knee OA [22–24]. Although RCTs examining the effects of combination therapy on physical activity have been conducted, no meta-analysis has been conducted to integrate these results.

Pain frequently occurs in patients with hip or knee OA who have not undergone surgical treatment. Exercise and educational interventions are applied in cases where pain is associated with decreased physical activity in hip or knee OA. Previous meta-analyses have shown inconsistent results regarding the effects of exercise or educational intervention on pain reduction [17, 25, 26]. One meta-analysis study has reported that a combination therapy of exercise and educational intervention reduces pain [25]; however, the control group included various interventions, such as exercise, educational intervention, acupuncture, and usual care, such that the exact effect of the combination therapy on pain reduction was not clear.

Therefore, we established the research question of this meta-analysis as follows: whether single or combination therapy with exercise and/or educational intervention is effective in improving physical activity and reducing pain in patients with hip or knee OA, compared to “pure” (no treatment) control or placebo patients.

## Methods

This systematic review was conducted in accordance with the Preferred Reporting Items for Systematic Reviews and Meta-Analyses [27, 28] and Cochrane Recommendation for Systematic Reviews [29, 30]. Bias assessment of included studies was performed using the Revised Cochrane risk-of-bias tool for randomized trials (RoB 2) according to the Cochrane Handbook for Systematic Reviews of Interventions [29–31]. The protocol of this systematic review was registered at the International Prospective Register of Systematic Reviews (registration code: CRD42020205804).

### Eligibility criteria

We established the research question using “Population, Intervention, Comparator, Outcome” (PICO) framework. Studies were included in the review if they: (1) met the PICO framework: (a) population: people with hip /knee OA of any severity and duration without hip /knee joint replacement, diagnosed by a doctor; (b) intervention: exercise, educational interventions, or both; (c) control: a pure control or placebo; (d) outcome measures: physical activity and pain; (2) were written in English; (3) were published as RCTs in peer-reviewed journals up until April, 2022; and (4) were available in full-text versions. Studies that newly applied analgesics after onset of the intervention as one of the coping mechanisms were excluded.

### Information sources and search strategy

According to the PRISMA-S [32], electronic database searches were performed in MEDLINE (PubMed), ProQuest, Scopus, and the Physiotherapy Evidence Database (PEDro), from the earliest records through December 31, 2020, and were later updated through April 30, 2022. The initial search concluded August 8, 2021. Two additional meta-analyses were identified by another reviewer in November, 2021, and we rechecked the databases for articles published through April, 2022.

The search combined key terms and synonyms for the categories of population (osteoarthritis), intervention (exercise, education), outcomes (pain, physical activity) and design (randomized controlled trials) based on the PICO framework. Synonyms within categories were combined with ‘OR’ and categories were then combined with the ‘AND’ operator. The actual search terms, search strategy and results of each database are shown in S1 File. The reference lists of the included studies and relevant systematic reviews were also examined for potential studies to include.

### Selection process

Four independent reviewers screened the titles and abstracts of all the retrieved citations for eligibility. Full-text articles were searched and reviewed to determine whether they met the inclusion criteria if there was insufficient information in the abstract and title to make a decision. The final inclusion of articles was based on an assessment by two independent reviewers, with disagreements resolved through a consensus or by bringing in another independent reviewer.

### Data collection process

Initially, the data were independently extracted by two investigators. The following data were extracted from each study: author/year, patient characteristics, intervention (methods, durations, and frequencies), time points, and outcomes. When two different treatments were compared in the same study, both interventions were included as independent study outcomes for the purposes of the meta-analysis because the aim of this study was to compare the effects of various interventions. Primary and secondary outcomes were collected at baseline, at interventional endpoint, and at final follow-up, if measured. For each study analysis, we required the mean difference between the baseline and final data, as well as the baseline and follow-up, and the standard deviation of that difference for each intervention. When the required data were not described in the studies, we calculated the mean difference and standard deviation needed to perform the meta-analysis using the data provided in each study, as described previously [33, 34]. If the data were described in medians and quartiles, the article was considered unadoptable. Checks occurred during the extraction process for potential discrepancies by another two investigators.

### Data items for intervention

To address our main aim, we defined exercise as any type of land-based or aquatic-based exercise program, such as aerobic exercise, strengthening, stretching, mind-body exercise (yoga, tai chi, and qigong), and combined interventions using more than one of these exercises. Educational intervention was defined as some form of education, such as pain management and coping skills, discussion of findings on the importance of physical activity, and specific guidance on the management of OA symptoms. Educational intervention could be delivered via a range of modalities, including face-to-face communication, teleconferencing, internet-based sessions, and/or the use of other multimedia content. Cognitive behavioral therapy and behavioral management were also included as educational interventions.

### Data items for outcome measures

The primary outcome measure was physical activity, which was measured using either subjective or objective measures. Subjective measures included self-answered questionnaires, such as the Physical Activity Scale for the Elderly, International Physical Activity Questionnaire, and Short Questionnaire to Assess Health-enhancing Physical Activity. Objective measures were obtained using a pedometer and activity tracker, with indicators such as the number of daily steps, activity duration, and actigraphy. The secondary outcome measure was pain relief using the following outcomes: Western Ontario and McMaster Universities Osteoarthritis Index pain, visual analog scale, and numerical rating scale of pain during activity, knee injury and osteoarthritis outcome score / hip disability and osteoarthritis outcome score, and the Japanese Knee Osteoarthritis Measure.

### Study risk of bias assessment

Three reviewers independently performed a quality assessment of each study using RoB 2 [31] according to the Cochrane Handbook for Systematic Reviews of Interventions [29, 30]. Risk of bias was assessed for each outcome individually. Five components of bias were evaluated: bias arising from the randomization process; bias due to deviations from intended interventions: bias due to missing outcome data; bias in measurement of the outcome; and bias in selection of the reported result [35]. Bias in selection of the reported result was assessed directly, as described in the guidance [28]. The risk of bias was classified as low risk of bias, some concern, or high risk of bias for each item, and the overall risk of bias for each study was discussed.

### Effect measures and synthesis methods

We performed the meta-analysis using Review Manager software, ver. 5.4 (Copenhagen: The Nordic Cochrane Center, The Cochrane Collaboration, 2020). We analyzed two time points: (1) endpoints, the data measured immediately after the final therapeutic intervention, and (2) follow-up, the final data measured during the follow-up period. To adopt an outcome indicator for both physical activity and pain, we utilized standard mean differences (SMDs) with 95% confidence intervals (CIs) to calculate pooled estimates for all comparisons. Continuous outcomes were analyzed using the random-effects model to calculate the weighted mean difference and 95% confidence interval, which were visualized in forest plots. Since the random-effects model incorporates both within- and between-study variance, it is better suited than the fixed-effects model. The treatment effects were further classified as small (< 0.20), moderate (0.21 to 0.79), and large (> 0.80) according to Cohen’s criteria [36]. Heterogeneity was investigated using the chi square test. In cases of severe heterogeneity, additional analyses were performed when the outliers were excluded. The statistical significance was set at *P* < 0.05.

### Reporting bias and certainty assessments

Reporting bias was assessed directly as described in the guidance [28]. If there were more than ten studies included in a single comparison, reporting bias was assessed using Funnel plots. The Grading of Recommendations, Assessment, Development, and Evaluation (GRADE) approach was used to interpret and evaluate the quality of evidence [37]. The methods and recommendations described in the Cochrane handbook and by the GRADE working group were used to assess the quality of the body of evidence using five domains: risk of bias [38], inconsistency [39], indirectness of evidence [40], imprecision of effect estimates [41], and potential reporting bias [42]. The strength of evidence was classified into four categories: high, moderate, low, or very low. The GRADEpro Guideline Development Tool (McMaster University, 2021, developed by Evidence Prime, Inc.) was used to evaluate the overall quality of evidence.

## Results

### Study selection

**Fig 1:**
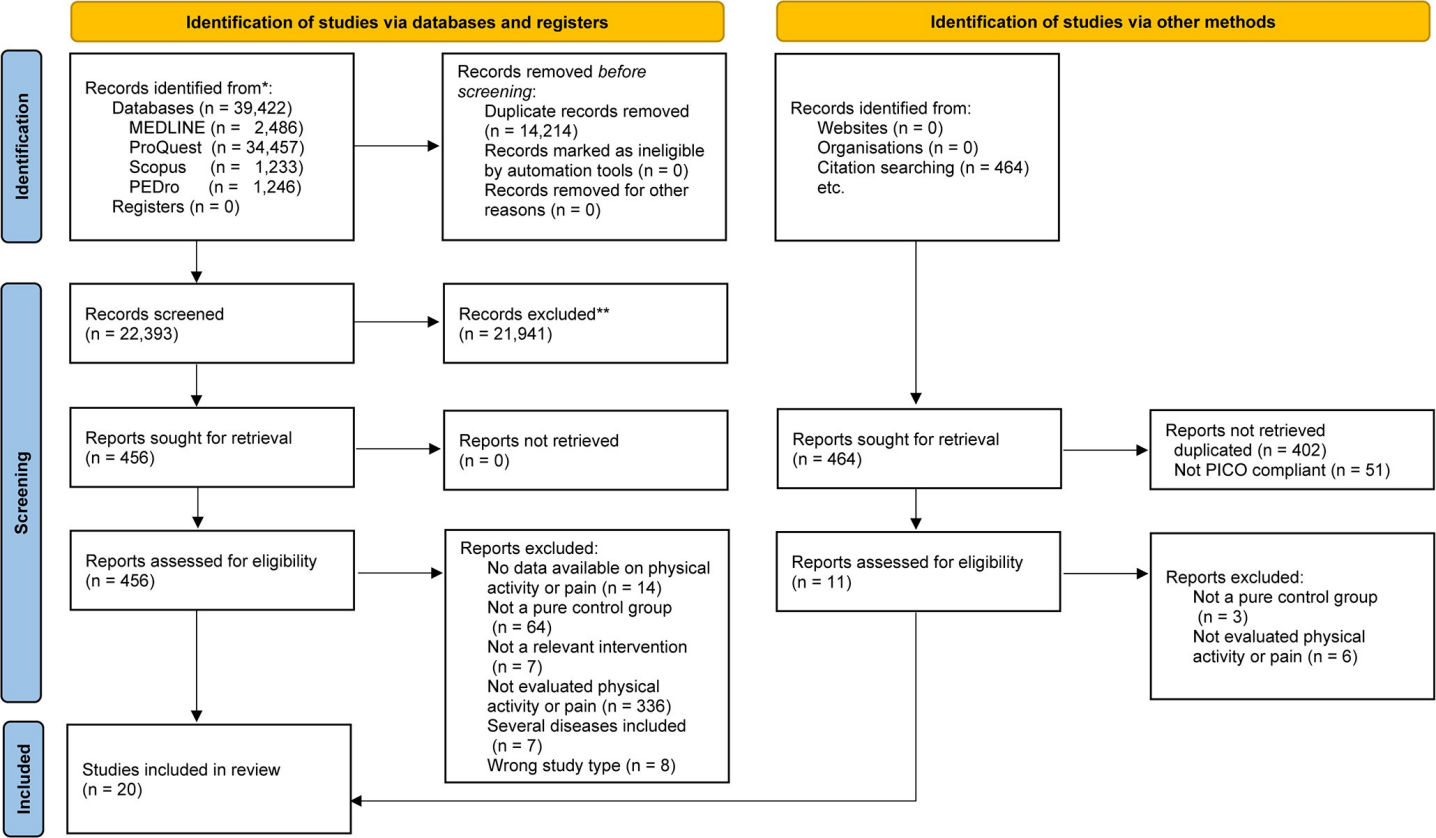
PRISMA 2020 flow diagram for new systematic reviews which included searches of databases, registers and other sources. *Consider, if feasible to do so, reporting the number of records identified from each database or register searched (rather than the total number across all databases/registers). **If automation tools were used, indicate how many records were excluded by a human and how many were excluded by automation tools. *From*: Page MJ, McKenzie JE, Bossuyt PM, Boutron I, Hoffmann TC, Mulrow CD, et al. The PRISMA 2020 statement: an updated guideline for reporting systematic reviews. BMJ 2021;372:n71. doi: 10.1136/bmj.n71. For more information, visit: http://www.prisma-statement.org/.

Fig 1 illustrates the different stages of the search and selection of studies included in our review. The initial search of the four electronic databases identified 39,886 titles and abstracts, of which 481 were retrieved for a full-text review. When the exclusion criteria were applied, 20 studies satisfied the criteria to be included in this review [22–24, 43–59]. The main reasons for exclusion were as follows: (1) no reports of outcomes of physical activity or pain and (2) the absence of a pure control group (S3 File).

### Study characteristics

The characteristics of the participants and the interventions applied in the 20 studies are summarized in Table 1. The sample sizes of the included studies ranged from 31 to 393 patients. A total of 16 studies recruited participants with knee OA [22, 23, 43, 44, 46, 48, 50–59]; one study recruited participants with hip OA [44]; and three studies recruited participants with both knee and hip OA [24, 47, 49]. The interventions to promote physical activity varied across the included studies: seven studies investigated exercise programs, such as internet-based exercise programs, walking programs, home exercise programs, manual therapy, and aquatic therapy [43, 45, 47, 54, 56, 57, 59]; eight studies investigated educational programs implementing motivational interviewing, educational sessions, individual counseling, self-monitoring and goal setting, and/or CBT programs [44, 46, 48, 50–53, 55]; and five studies included a combination program of exercise and educational interventions [22–24, 49, 58]. Regarding the assessment method, six studies used subjective outcomes of physical activity [43, 45, 47, 48, 54, 59], and 15 studies used objective outcomes [22–24, 44–46, 49–53, 55–58].

**Table 1:** Characteristics of the studies’ participants and interventions.

Study author, year (ref.)	Condition and patient characteristics 1) subject; 2) number of participants; 3) duration of OA; 4) severity of OA; 5) age	Intervention	1) Intervention period 2) frequency	Outcome of 1) physical activity, 2) pain, and 3) time points
Allen KD et al. 2018 [43]	1) Knee OA 2) 350 patients (group 1 = 140 (female = 98), group 2 = 142 (female = 100), group 3 = 62 (female = 53)) 3) 13.1 years 5) group 1, 65.3 ± 11.1 years; group 2, 65.7 ± 10.3 years; group 3, 64.3 ± 12.2 years	Group 1 (Ex): Internet-based exercise training program (Encouraged to complete strengthening and stretching exercises at least 3 times per week and to engage in aerobic exercises daily, or as often as possible) Group 2 (Ex): Home exercise program and up to eight 1 h supervised physical therapy sessions (home exercise program including strengthening, stretching/range of motion, and aerobic exercises, activity pacing) Group 3 (Con): Waiting list.	1) 4 months 2) group 1 at least 3 times / week, group 2 not listed	1) PASE 2) WOMAC 3) BL, 4 months, 12 months (f)
Bartholody C et al. [44]	1) Knee OA 2) 38 patients (group 1 = 19 (female = 15), group 2 = 19 (female = 14)) 3) no listed 5) group 1, 68.0 ± 7.3 years; group 2, 62.0 ± 9.7 years	Group 1 (Edu): Motivational text message (Information and general advice about the importance of performing daily physical activity) Group 2 (Con): No intervention	1) 6 weeks 2) 3 times / week	1) Activity time 2) KOOS 3) BL, 6 weeks
Bennell KL et al. [45]	1) Hip OA 2) 102 patients (group 1 = 49 (female = 26), group 2 = 53 (female = 36) 4) grade 2/3/4: group 1 24/11/14; group 2 27/14/12 5) group 1, 68.0 ± 7.3 years; group 2, 62.0 ± 9.7 years	Group 1 (Ex): Home exercise (strengthening, stretching, range of motion, functional balance, and gait drills), education, and advice Group 2 (Con): Inactive ultrasound and inert gel	1) 12 weeks 2) group 1 4 times / week, group 2 3 times / week	1) PASE, Steps/day 2) VAS, KOOS 3) BL, 13 weeks, 36 weeks (f)
Gilbert AL et al. 2018 [46]	1) Knee OA 2) 155 patients (group 1 = 76 (female = 57.89%), group 2 = 79 (female = 62.03)) 4) grade 2/3/4 (%): group 1 50.0/27.6/22.4; group 2 58.2/26.6/15.2 5) group 1, 61.4 ± 13.3 years; group 2, 64. 8 ± 12.4 years	Group 1 (Edu): Motivational interviewing (45–60 minutes; individual counseling based on motivational interviewing, individualized goal setting, and tailored strategies for increasing physical activity and monitoring progress) Group 2 (Con): Physician-conducted physical activity counseling session only	1) 24 months 2) (in first year) 3, 6, and 12 months, (in second year) every 6 months	1) Activity time 2) WOMAC 3) BL, 3 months, 6 months, 12 months, 24 months
Hinman RS et al. [47]	1) Hip/Knee OA 2) 71 patients (group 1 = 36 (female = 24), group 2 = 35 (female = 24)) 3) group 1 8.0 years, group 2 8.0 years 5) group 1, 63.3 ± 9.5 years; group 2, 61.5 ± 7.8 years	Group 1 (Ex): Aquatic physical therapy program (Encouraged to continue independent aquatic physical therapy 2 times weekly and recorded in a logbook the sessions of independent aquatic physical therapy) Group 2 (Con): Usual activities and medication regimen	1) 6 weeks 2) 2 times / week	1) PASE 2) VAS, WOMAC 3) BL, 6 weeks, 12 weeks (f)
Hinman RS et al. 2020 [48]	1) Knee OA 2) 71 patients (group 1 = 87 (female = 55), group 2 = 88 (female = 55)) 3) group 1 10.0 years, group 2 9.0 years 5) group 1, 62.4 ± 9.1 years; group 2, 62.5 ± 8.1 years	Group 1 (Edu): In addition to Group 2, 5–10 consultations with a physiotherapist using telephone planning for “hard” to “very hard” levels of physical activity. Group 2 (Con): Provided information about OA; treatments and self-management strategies; community resources; assistance navigating services; emotional support and care escalation, that was delivered once by nurses.	1) 6 months 2) 5–10 times / 6 months	1) PASE 2) NRS, WOMAC 3) BL, 6 months, 12 months (f)
Hughes SL et al. 2004 [49]	1) Hip/Knee OA 2) 150 patients (group 1 = 80 (female = 81.0%), group 2 = 70 (female = 87.1%)) 4) ACR class (grade 1/2/3) (%): group 1, 23.0/66.2/10.8, group 2, 21.0/66.1/12.9 5) group 1, 73.5 ± 6.8 years; group 2, 73.7 ± 6.3 years	Group 1 (Ex + Edu): Fit and Strong intervention (Resistance training for the lower extremities and trunk, 30 min fitness walking (intensity is to 40% to 60% of maximum heart rate or 13 to 15 on the Borg Scale) + Education (30 min group-discussion-educational component: self-efficacy for exercise, exercise adherence, for manage pain and other arthritis-related symptoms, goal settings and feedback to participants on progress made toward the achievement of these goals) Group 2 (Con): The Arthritis Helpbook, a list of exercise programs	1) 8 weeks 2) 90 min / 3 times / week	1) Time spent walking 2) WOMAC 3) BL, 2 months, 6 months (f)
Hughes SL et al. 2006 [24]	1) Hip/Knee OA 2) 215 patients (group 1 = 115 (female = 80.6%), group 2 = 100 (female = 85.9%)) 4) ACR class (grade 1/2/3) (%): group 1 22.6/64.5/12.9, group 2 22.2/64.2/13.6 5) group 1, 73.3 years; group 2, 73.4 years	Group 1 (Ex + Edu): Fit and Strong intervention (Resistance training for the lower extremities and trunk, 30 minutes’ fitness walking (intensity is to 40% to 60% of maximum heart rate or 13 to 15 on the Borg Scale) + Education (30 min group-discussion-educational component: self-efficacy for exercise, exercise adherence, for manage pain and other arthritis-related symptoms, goal settings and feedback to participants on the progress made toward the achievement of these goals) Group 2 (Con): The Arthritis Helpbook, a list of exercise programs	1) 8 weeks 2) 90 min / 3 times / week	1) Time spent walking 2) WOMAC 3) BL, 2 months, 6 months (f), 12 months (f)
Li LC et al. 2017 [50]	1) Knee OA 2) 34 patients (group 1 = 17 (female = 14), group 2 = 17 (female = 14)) 4) Diagnosis OA: yes: 20 (59%), No, but met the "likely OA" criteria: 14 (41%) 5) 55.5 ± 8.6 years; (group 1, 52.3 ± 9.7 years; group 2, 58.7 ± 6.0 years)	Group 1 (Edu): 1.5-h session; standardized group education session about physical activity (the benefits of physical activity, the detrimental effects of sedentary behavior, ways to be active without aggravating OA symptoms), individual weekly activity counseling with a PT via telephone (identify activity goals, develop an action plan, identify barriers and solutions, rate their confidence in executing the plan), and tracking the participant’s physical activity behavior by using Fitbit Flex Group 2 (Con): Waiting list	1) 2 months 2) 4 weekly 20 min telephone call	1) MVPA 2) KOOS 3) BL, 1 months, 2 months
Li LC et al. 2018 [51]	1) Knee OA 2) 61 patients (group 1 = 30 (female = 22), group 2 = 31 (female = 28)) 4) Diagnosis OA: yes: 52 (85%), No, but met the "likely OA" criteria: 9 (15%) 5) 61.7 ± 8.9 years; (group 1, 61.3 ± 9.4 years; group 2, 62.1 ± 8.5 years)	Group 1 (Edu): 1.5-h session; standardized group education session about physical activity (the benefits of physical activity, the detrimental effects of sedentary behavior, ways to be active without aggravating OA symptoms), individual weekly activity counseling with a PT via telephone (identify activity goals, develop an action plan, identify barriers and solutions, rate their confidence in executing the plan), and tracking the participant’s physical activity behavior by using Fitbit Flex Group 2 (Con): Waiting list	1) 2 months 2) 4 weekly 20 min telephone call	1) MVPA 2) KOOS 3) BL, 1 months, 2 months, 4 months (f), 6 months (f)
Li LC et al. 2020 [52]	1) Knee OA 2) 51 patients (group 1 = 26 (female = 23), group 2 = 25 (female = 19)) 4) Diagnosis OA: yes: 37 (73%), No, but met the "likely OA" criteria: 14 (27%) 5) 64.9 ± 8.5 years; (group 1, 65.0 ± 8.0 years; group 2, 64.8 ± 9.0 years)	Group 1 (Edu): 3 components; an in-person session with 20 min of group education and 30 min of individual counseling with a PT, use of pedometer (Fitbit Flex-2 wristband), PT counseling by phone to review physical activity goals (20–30 min). During weeks 1 to 8, PT remotely reviewed the participants’ progress and counseled them to modify their physical activity goals via 4 biweekly phone calls. During weeks 9 to 12, the participants continued using their Fitbit without counseling. Group 2 (Con): Waiting list	1) 12 weeks 2) 4 biweekly telephone call	1) Daily steps, MVPA 2) KOOS 3) BL, 13 weeks, 26 weeks (f), 39 weeks (f)
Moseng T et al. 2020 [22]	1) Knee OA 2) 393 patients (group 1 = 284 (female = 211), group 2 = 109 (female = 68)) 3) no listed 5) group 1 63.0 ± 10.0 years; group 2 65.0 ± 10.0 years	Group 1 (Ex + Edu): 3-hour group-based education program, focused on knowledge of OA, recommend treatments, emphasizing the importance of exercise. 1 hour group exercise with 5–10 patients. 2–4 sets with 8–12 repetitions of 60–70% of 1 RM were set up. 30–60 minutes of walking and pedaling exercises at home were also recommended. Group 2 (Con): Usual care excluding individual exercise and education program.	1) 8–12 weeks 2) 2 times / weeks	1) sitting time 2) NRS, KOOS, HOOS 3) BL, 3 months, 6 months (f)
Murphy SL et al. 2018 [53]	1) Knee OA 2) 57 patients (group 1 = 38 (female = 24), group 2 = 19 (female = 11)) 3) no listed 5) 63.5 ± 8.3 years (group 1 64.8 ± 8.0 years; group 2 60.7 ± 8.5 years)	Group 1 (Edu): 1-hour session with occupational therapist (explanation of the program’s focus on lifestyle changes to help manage osteoarthritis symptoms through self-monitoring and goal setting,) and online program about CBT program (exercise, sleep, hygiene, pleasant activity scheduling, relaxation, activity pacing, problem solving, wrap-up session that focused on further goal attainment) Group 2 (Con): Usual osteoarthritis care	1) 6 months 2) 8 times / week	1) average activity counts 2) BPI 3) BL, 6 months
Rewald S et al. 2020 [54]	1) Knee OA 2) 102 patients (group 1 = 55 (female = 70.9%), group 2 = 47 (female = 51.1%)) 4) K/L grade: group 1 2.0 ± 0.6, group 2 2.0 ± 0.5 5) group 1, 59.0 ± 9.5 years; group 2, 61.0 ± 7.4 years	Group 1 (Ex). The participants rowed the aqua bike in an upright position in the pool and adjusted their legs to be soaked during the entire row. The participants were immersed in warm water (32°C) between the xiphoid process and the first rib. The participants also incorporated an unsaddled posture, leg exercises, and upper body exercises. Group 2 (Con): Usual care, follow-up of treatment received outside the study is not prohibited.	1) 12 weeks 2) 45 min / 2 times / week	1) SQUASH 2) KOOS, NRS 3) BL, 12 weeks, 24 weeks (f)
Schlenk EA et al. 2020 [23]	1) Knee OA 2) 182 patients (group 1 = 91 (female = 67), group 2 = 91 (female = 66)) 3) group 1 11.7 ± 9.9 years, group 2 11.3 ± 9.2 years 5) 64.7 ± 8.1 years; (group 1, 64.5 ± 8.5 years; group 2, 65.0 ± 7.8 years)	Group 1 (Ex + Edu): 6 weekly 60–65 min individual face-to-face sessions including graduated LEE, and progressive fitness walking. Nine biweekly 15–20 min telephone sessions with the licensed registered nurse for ongoing goal setting and support, which were conducted in the project office; and daily e-diary for self-monitoring physical activity during the 6-month intervention. Group 2 (Con): Usual care and 15 sessions of 15–20 min telephone sessions. 15 sessions covered health topics from the National Institute on Aging website that avoided any mention of knee OA and their management, including physical activity.	1) 6 months 2) 6 weekly face-to-face sessions and Nine biweekly 15–20 min telephone call	1) activity time 2) WOMAC 3) BL, 6 months, 12 months (f)
Shahine NF et al. 2020 [55]	1) Knee OA 2) 66 patients (group 1 = 33 (female = 21), group 2 = 33 (female = 16)) 3) (less than 5 years/ 5–10 years/ +10 years): group 1 16/10/7, group 2 14/13/6 5) group 1, 65.6 ± 5.0 years; group 2, 66.9 ± 5.8 years	Group 1 (Edu): Pedometer self-monitoring, aerobic weekly step count goals and weekly telephone follow ups. Educational booklet used for improving the patient’s knowledge level in four sessions. The patients received their individualized daily step count goals every week to increase gradually by 10% of baseline steps/d for weeks 2–12. Patients received a weekly telephone call of duration10-15 min for feedback and providing new step count goals. Group 2 (Con): Usual care	1) 12 weeks 2) 10–15 minutes telephone / week, 4 session for education with booklet	1) Daily steps 2) WOMAC 3) BL, 12 weeks
Vincent KR et al. 2020 [56]	1) Knee OA 2) 88 patients (group 1 = 17 (female = 11), group 2 = 19 (female = 13), group 3 = 17 (female = 11)) 3) group 1 7.8 ± 8.1 years; group 2 12.8 ± 11.9 years; group 3 7.9 ± 8.1 years 5) 68.3 ± 6.4 years (group 1, 69.5 ± 6.5 years; group 2, 66.8 ± 5.4 years; group 3, 68.6 ± 7.1 years)	Group 1 (Ex): Concentrically focused resistance training of 12 repetitions with 60% 1 RM. One set of each of the following exercises was completed during each session: leg press, knee flexion, knee extension, calf press, chest press, seated row, shoulder press, and biceps curl. The resistance load was raised for the set to keep the rating of perceived exertion value at approximately 17–18 of 20 points for each exercise over the study duration. Group 2 (Ex): Eccentrically focused resistance training enhanced eccentric training continually performs the eccentric muscle action with the equivalent of the 1RM and to sequentially reduce the load to 60% of 1RM for the concentric muscle action. Group 3 (Con): Usual activity	1) 4 months 2) 2 times / week	1) Daily steps, activity time 2) NRS 3) BL, 4 months
Waller B et al. 2017 [57]	1) Knee OA 2) 87 patients; all women (group 1 = 43, group 2 = 44) 4) grade 1/2: group 1 23/20; group 2 24/20 5) group 1, 63.8 ± 2.4 years; group 2, 63.9 ± 2.4 years	Group 1 (Ex): Aquatic resistance training sessions (three resistance levels (barefoot, small resistance fins, large resistance boots)) Training intensity was set at as “hard and fast as possible” Group 2 (Con): Usual care (continue their usual leisure time activities, participate in two sessions consisting of 1 h of light stretching, relaxation, and social interaction)	1) 16 weeks 2) 1 hour / 3 times / week	1) LTPA 2) KOOS 3) BL, 4 months, 16 months (f)
Wallis JA et al. 2017 [58]	1) Knee OA 2) 46 patients (group 1 = 23 (female = 9), group 2 = 23 (female = 11)) 4) grade 3/4: group 1 1/21; group 2 2/21 5) group 1, 68.8 ± 8.0 years; group 2, 67.0 ± 7.0 years)	Group 1 (Ex + Edu): Walking (at least moderate intensity: determined by the Rate of Perceived Exertion Scale (0 to 10) where level 3 = “I am still comfortable but am breathing a little harder”), behavioral change techniques and strategies (①planning session with a physiotherapist (up to 30 min to plan the location, day and time of day for each walk) ②regular physiotherapy supervision and monitoring each week (one-to-one supervised walking sessions or group supervised walking sessions based on patient preference, and regular phone calls or SMS reminders) ③wearing a pedometer and recording the number of steps taken and time spent walking during each session in a logbook ④engaging social supports such as walking with a friend, family member or other research participants) + usual care Group 2 (Con): Non-operative management to manage pain and symptoms including pharmacological and non-pharmacological interventions	1) 12 weeks 2): at least 10 min / time, 70 min / week	1) Steps, Walking time 2) NRS, WOMAC 3) BL, 13 weeks
Wortley M et al. 2013 [59]	1) Knee OA 2) 31 patients (group 1 = 13 (female = 9), group 2 = 12 (female = 9), group 3 = 6 (female = 4)) 4) K/L grade (median(range)): group 1 2 (2); group 2 3 (3); group 3 2 (1) 5) group 1, 69.5 ± 6.7 years; group 2, 68.1 ± 5.3 years; group 3, 70.5 ± 5.0 years	Group 1 (Ex): Open-kinetic chain resistance training program including seated leg extension, standing hamstring curl, straight leg raise, standing hip abduction, standing hip adduction, standing hip flexion, standing calf raise, which started with either a 5 lb. or 10 lb. ankle weight and progressed from two sets of eight repetitions to three sets of 12 repetitions during the first 6 weeks, and were allowed to increase the weight as needed during the final 4 weeks. Group 2 (Ex): 1-hour training of 12 basic movements adapted from the Yang Style Tai Ji. The program began by learning the first two movements during the first session, and then adding a new movement during each session for the first 5 weeks. Group 3 (Con): Usual physical activity	1) 10 weeks 2) 1 hour/ section; 2 times / week	1) PASE 2) WOMAC 3) BL, 6 months

Abbreviations. ref, reference number; OA, osteoarthritis; Ex, exercise; Edu, educational intervention; Ex + Edu, Combination therapy of exercise and educational intervention; Con, control; ACR, American College of Rheumatology; K/L, Kellgren-Lawrence; PT, physical therapy; RM, repetition maximum; CBT, cognitive behavioral therapy; LEE, lower extremity exercise; BL, baseline; PASE, physical activity scale for Elderly; WOMAC, Western Ontario and McMaster Universities Osteoarthritis Index; KOOS, knee injury and osteoarthritis outcome score; VAS, visual analog scale; NRS, numerical rating scale; MVPA, moderate to vigorous physical activity; HOOS, hip disability and osteoarthritis outcome score; BPI, brief pain inventory; SQUASH, short questionnaire to assess health-enhancing physical activity; LTPA, average monthly leisure time physical activity

### Risk of bias in studies

**Fig 2:**
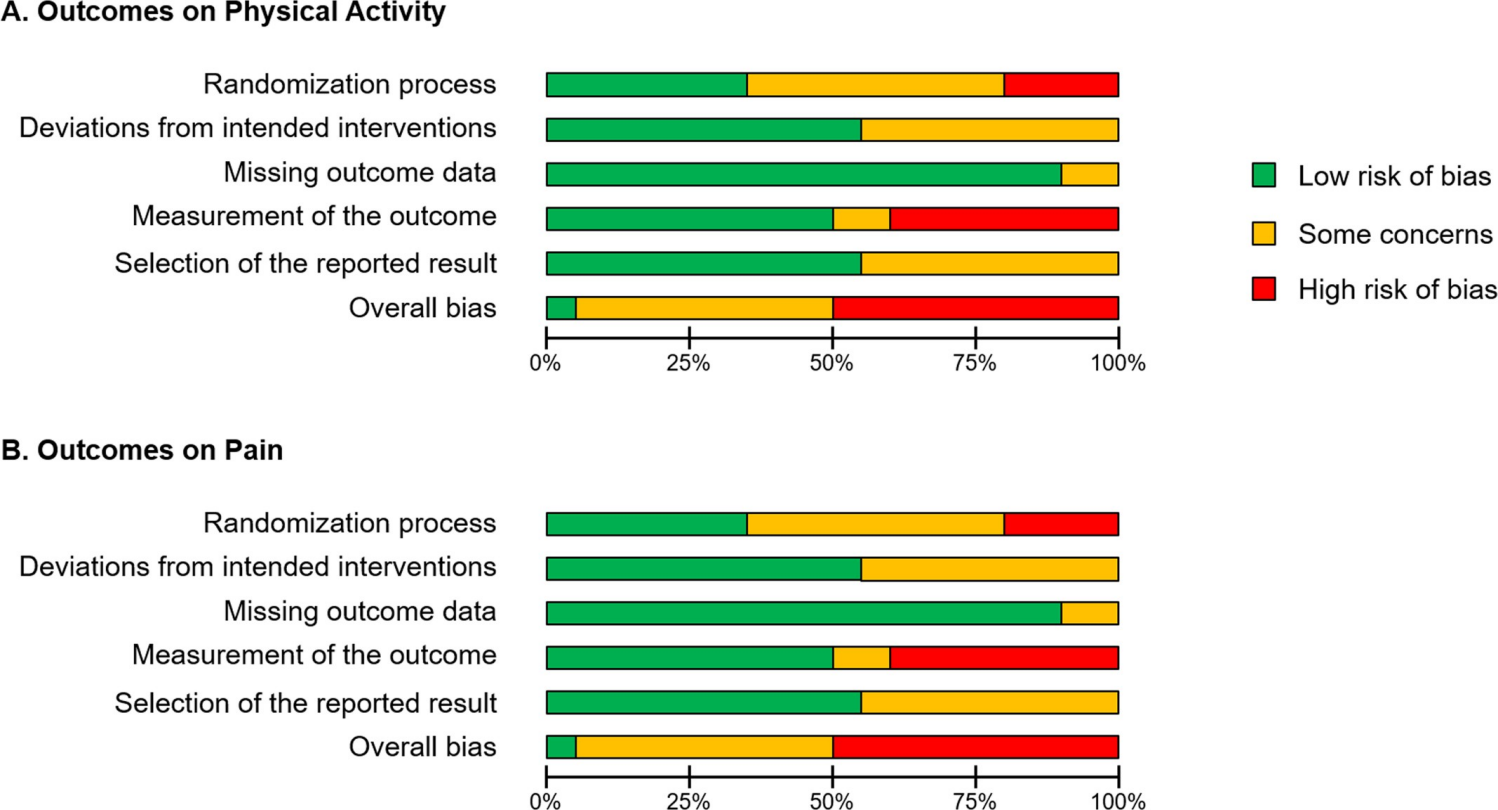
Summary in risk of bias 2. (A) Outcomes on physical activity. (B) Outcomes on pain. Green, Low risk of bias; Yellow, Some concerns; Red, High risk of bias.

Fig 2 provides a summary of the RoB 2 results for the 20 included studies. In addition, S2 File shows the justification for each rating in each outcome of each study. Although we scored each outcome respectively, the RoB 2 results were in perfect agreement. In the bias arising from the randomization process, seven studies were identified as having low risk of bias [46–48, 52, 56–58], and four as having high risk of bias [45, 49, 55, 59]. In the bias due to deviations from intended interventions, 11 studies were rated as low risk of bias [22, 23, 43, 48, 50–52, 54, 55, 58, 59]. In the bias due to missing outcome data, 18 studies were identified as low risk of bias [23, 24, 43–53, 55–59]. In the bias in measurement of the outcome, 10 studies were identified as having low risk of bias [23, 43, 45–48, 53, 54, 56, 58], and eight as having high risk of bias [24, 44, 49–52, 55, 57]. In the bias in selection of the reported result, 11 studies were identified as having low risk of bias [22, 43–46, 48, 50–52, 54, 56]. Only one study [48] showed low risk of bias in all five components while 10 studies showed a high risk of bias in at least one component [24, 44, 45, 49–52, 55, 57, 59].

### Result of individual studies and synthesis

#### Effect of exercise on physical activity

**Fig 3:**
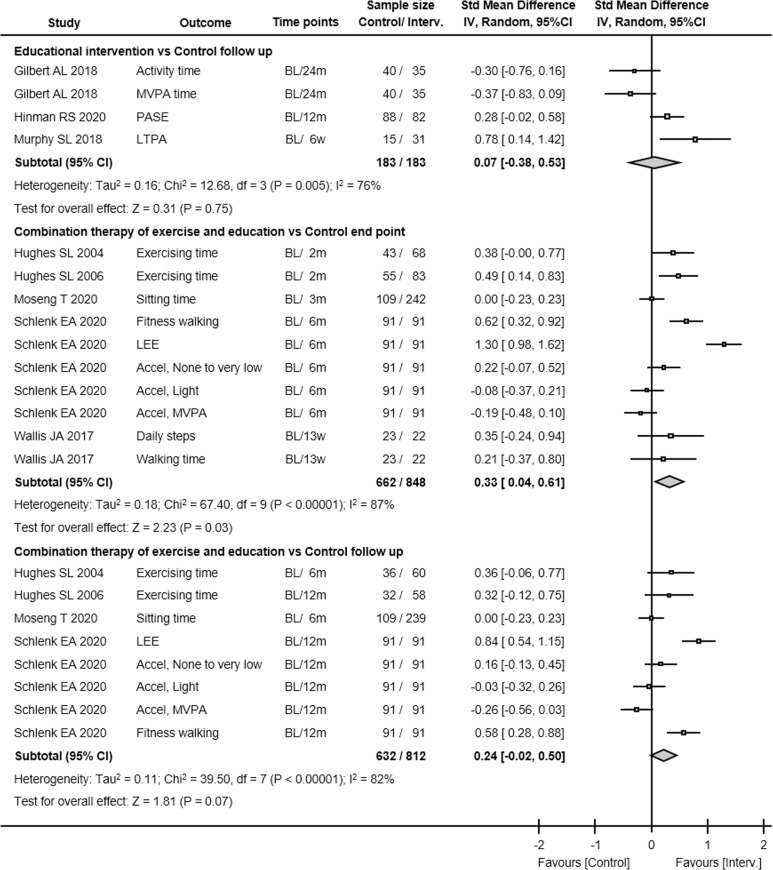
The mean difference and 95% CI values in physical activities. Results from each study. Interv., intervention; Std Mean Difference, standardized mean difference; IV, inverse variance; 95% CI, 95% confidence interval; PASE, Physical Activity Scale for the Elderly; SQUASH, short questionnaire to assess health-enhancing physical activity; LTPA, average monthly leisure time physical activity; MVPA, moderate to vigorous physical activity; METs, metabolic equivalents; LEE, Lower-extremity exercise; Accel, accelerometer.

Seven studies with 1,116 participants evaluated the effect of exercise interventions on physical activity levels immediately post-intervention. There was low GRADE evidence that exercise interventions were not associated with differences in physical activity at this end point (SMD 0.11, 95% CI -0.04 to 0.25, P = 0.20, I^2^ 27%; Fig 3). Although there were not serious issues in quality assessment except for risk of bias, the evidence GRADE was low (Table 2). This comparison included both subjective and objective outcomes, but no significant differences in results were found when subgroup analysis (objective vs. subjective) was performed. In addition, there were no significant differences for end point physical activity among the intervention types.

**Table 2:** Evidence for outcome measures. SMD, standardized mean difference; 95% CI, 95% confidence interval.

Comparison No, studies (ref.)	Quality assessment				No. patients		Effect, SMD (95% CI)	Quality
	Risk of bias	Inconsistency	Indirectness	Imprecision	Intervention	Control		
**Physical Activity, Ex vs Con**								
End point 7 (43, 45, 47, 54, 56, 57, 59)	Very Serious	Not Serious (I^2^ = 27%)	Not Serious	Not Serious	668	528	0.11 (-0.04 to 0.25)	⊕⊕○○ Low
Follow up 4 (43, 45, 54, 57)	Very Serious	Not Serious (I^2^ = 0%)	Not Serious	Not Serious	401	301	0.10 (-0.05 to 0.25)	⊕⊕○○ Low
**Physical Activity, Edu vs Con**								
End point 7 (44, 46, 48, 50–52, 55)	Very Serious	Very Serious (I^2^ = 70%)	Not Serious	Not Serious	474	494	0.24 (-0.00 to 0.48)	⊕○○○ Very Low
Follow up 3 (46, 48, 53)	Very Serious	Very Serious (I^2^ = 76%)	Not Serious	Serious	183	183	0.07 (-0.38 to 0.53)	⊕○○○ Very Low
**Physical Activity, Ex + Edu vs Con**								
End point 5 (22–24, 49, 58)	Very Serious	Very Serious (I^2^ = 87%)	Not Serious	Not Serious	848	662	**0.33 (0.04 to 0.61)**	⊕○○○ Very Low
Follow up 4 (22–24, 49)	Very Serious	Very Serious (I^2^ = 82%)	Not Serious	Not Serious	812	632	0.24 (-0.02 to 0.50)	⊕○○○ Very Low
**Pain, Ex vs Con**								
End point 7 (43, 45, 47, 54, 56. 57, 59)	Very Serious	Not Serious (I^2^ = 0%)	Not Serious	Not Serious	746	609	**-0.25 (-0.36 to -0.14)**	⊕⊕○○ Low
Follow up 4 (43, 45, 54, 57)	Very Serious	Not Serious (I^2^ = 0%)	Not Serious	Not Serious	464	341	**-0.15 (-0.29 to -0.01)**	⊕⊕○○ Low
**Pain, Edu vs Con**								
End point 7 (44, 46, 48, 50–52, 55)	Very Serious	Very Serious (I^2^ = 83%)	Not Serious	Not Serious	436	443	**-0.39 (-0.74 to -0.05)**	⊕○○○ Very Low
Follow up 3 (46, 48, 53)	Very Serious	Not Serious (I^2^ = 0%)	Not Serious	Not Serious	327	319	-0.12 (-0.28 to 0.03)	⊕⊕○○ Low
**Pain, Ex + Edu vs Con**								
End point 5 (22–24, 49, 58)	Very Serious	Not Serious (I^2^ = 0%)	Not Serious	Not Serious	569	344	**-0.15 (-0.29 to -0.02)**	⊕⊕○○ Low
Follow up 4 (22–24, 49)	Very Serious	Not Serious (I^2^ = 0%)	Not Serious	Not Serious	492	268	**-0.18 (-0.33 to -0.03)**	⊕⊕○○ Low

Regarding follow-up, four studies with 702 participants evaluated the effect of exercise interventions on physical activity levels at the end of the follow-up. There was low GRADE evidence that exercise was not associated with differences in physical activity at follow-up (SMD 0.10, 95% CI -0.05 to 0.25, P = 0.20, I^2^ 0%). Because the risk of bias was defined as very serious, the GRADE evidence at follow-up was determined to be “low”.

#### Effect of educational intervention on physical activity

Seven studies with 968 participants evaluated the effect of educational interventions on physical activity levels immediately post-intervention. There was very low GRADE evidence that educational intervention was not associated with differences in physical activity (SMD 0.24, 95% CI -0.00 to 0.48, P = 0.05, I^2^ 70%; Fig 3). However, the authors concluded that the GRADE level was "very low" because of the significant risk of bias and severe inconsistencies (Table 2). In addition, although the outlier data [55] were excluded and heterogeneity remained at 2%, there were also no effects in the educational intervention on physical activity at the end point.

At the follow-up, three studies with 366 participants evaluated the effect of educational interventions on physical activity levels at the end of follow-up. There was very low GRADE evidence that educational intervention was not associated with differences in physical activity at follow-up (SMD 0.07, 95% CI -0.38 to 0.53, P = 0.75, I^2^ 76%). Because the risk of bias was defined as very serious and severe inconsistency was noted, the GRADE evidence at follow-up was determined “very low”.

#### Effect of combination therapy of exercise and educational intervention on physical activity

Five studies with 1,510 participants evaluated the effect of the combination of exercise and educational interventions on physical activity levels immediately post-intervention. The combination of exercise and educational interventions was associated with differences in physical activity (SMD 0.33, 95% CI 0.04 to 0.61, P = 0.03, I^2^ 87%; Fig 3), but the GRADE level was very low because very serious risks of bias and severe inconsistency were noted (Table 2).

At the follow-up, four studies with 1,444 participants evaluated the effect of the combined intervention of exercise and educational interventions on physical activity levels at the end of follow-up condition. There was evidence that combined intervention of exercise and educational interventions was not associated with differences in physical activity at follow-up (SMD 0.24, 95% CI -0.02 to 0.50, P = 0.07, I^2^ 82%), but the GRADE level was very low because the risk of bias was defined as very serious and severe inconsistency noted.

#### Effect of exercise on pain

**Fig 4:**
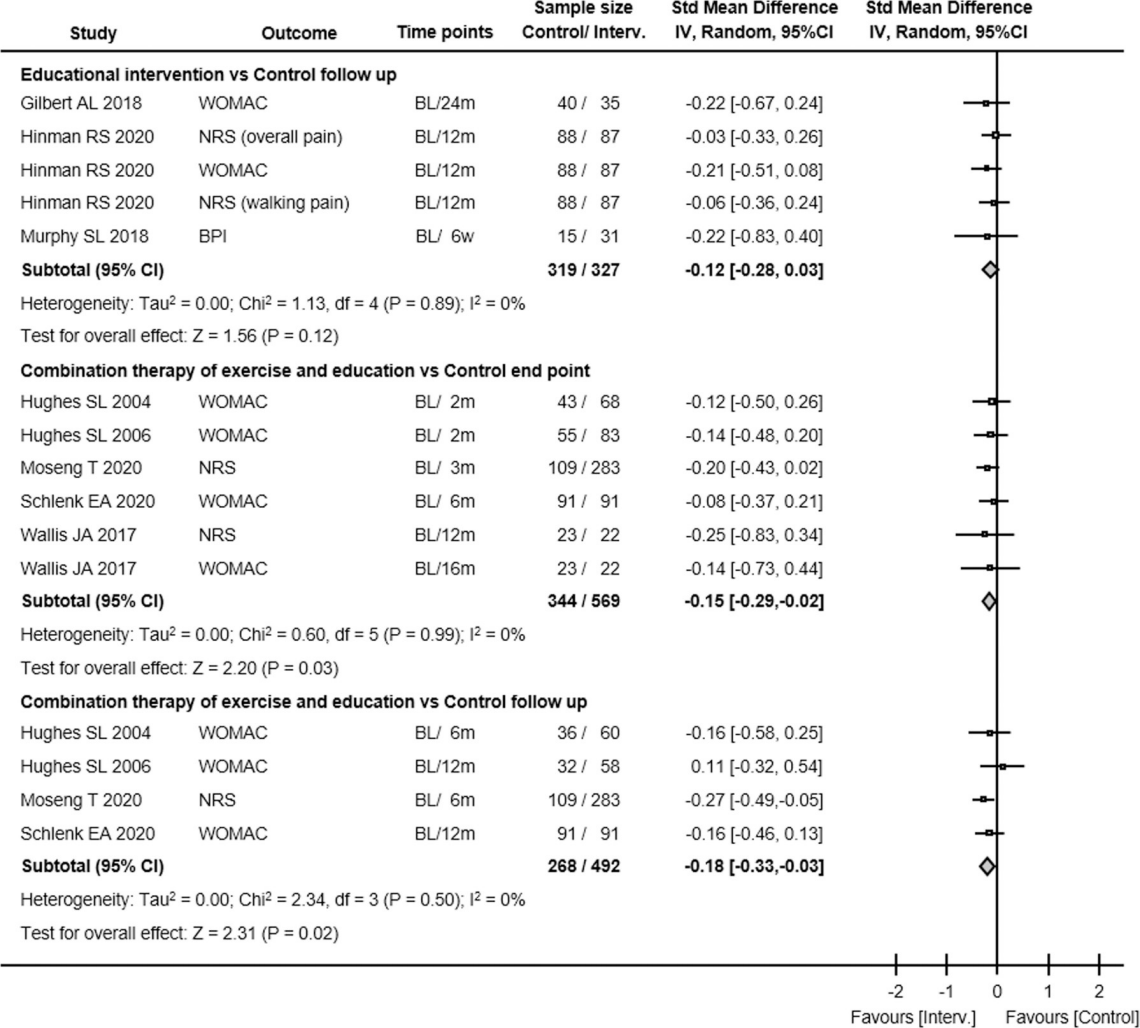
The mean difference and 95% CI values in pain. Results from each study. Interv., intervention; Std Mean Difference, standardized mean difference; IV, inverse variance; 95% CI, 95% confidence interval; WOMAC, Western Ontario and McMaster Universities Osteoarthritis Index; VAS, visual analogue scale; HOOS, Hip disability and Osteoarthritis Outcome Scale; KOOS, Knee injury and Osteoarthritis Outcome Scale; NRS, numerical rating scale; BPI, brief pain inventory.

Seven studies with 1,355 participants evaluated the effect of exercise interventions on pain immediately post-intervention. There was evidence that exercise interventions were associated with differences in pain (SMD -0.25, 95% CI -0.36 to -0.14, P < 0.01, I^2^ 0%; Fig 4) but the GRADE level was determined to be low because there was very serious risk of bias (Table 2).

At the follow-up, four studies with 805 participants evaluated the effect of exercise interventions on pain at the end of the follow-up condition. There was evidence that exercise was also associated with differences in pain at follow-up (SMD -0.15, 95% CI -0.29 to -0.01, P = 0.04, I^2^ 0%), but the GRADE level was low because the risk of bias was defined as very serious.

#### Effect of educational intervention on pain

Seven studies with 879 participants evaluated the effect of educational interventions on pain immediately post-intervention. There was evidence that educational interventions were associated with differences in pain (SMD -0.39, 95% CI -0.74 to -0.05, P = 0.03, I^2^ 83%; Fig 4) but the GRADE level was determined to be very low because very serious risk of bias and severe inconsistency were noted. Even with the exclusion of outlier data [55] and heterogeneity, the effects of educational intervention on pain at the end point were remained very low (Table 2).

Three studies with 646 participants evaluated the effect of educational interventions on pain at the end of follow-up condition. There was evidence that educational intervention was not associated with differences in pain at follow-up (SMD -0.12, 95% CI -0.28 to 0.03, P = 0.12, I^2^ 0%) but the GRADE level was low because the risk of bias was defined as very serious.

#### Effect of combination therapy of exercise and educational intervention on pain

Five studies with 913 participants evaluated the effect of the combination of exercise and educational interventions on pain immediately post-intervention. There was evidence that the combination of exercise and educational interventions were associated with differences in pain (SMD -0.15, 95% CI -0.29 to -0.02, P = 0.03, I^2^ 0%; Fig 4) but the GRADE level was low because a very serious risk of bias was identified (Table 2).

Four studies with 760 participants evaluated the effect of combined intervention of exercise and educational interventions on pain at the end of follow-up condition. There was evidence that combined intervention of exercise and educational interventions was associated with differences in pain at follow-up (SMD -0.18, 95% CI -0.33 to -0.03, P < 0.01, I^2^ 0%) but the GRADE level was low because the risk of bias was defined as very serious.

## Discussion

Exercise and education are non-pharmacological interventions recommended as first-line management strategies for patients with OA [19, 60]. However, it remains unknown whether intervention with exercise and/or education is effective at increasing physical activity. Therefore, we investigated the effectiveness of exercise and/or educational intervention for physical activity and pain in patients with nonsurgical OA using a meta-analysis method.

At the end point (immediately post-intervention), we found very low evidence that exercise intervention alone was not effective for activate physical activity levels. In addition, there were also very low evidence that exercise intervention alone cannot upregulate the physical activity levels at follow up. These results are similar to those of Oliveira CB et al. [20], which showed no effectiveness of exercise on improving physical activity. Looking at the extracted articles, an article investigating the effect of aquatic exercise [57] found an increase in physical activity. On the other hand, no increase in physical activity was observed in the other studies that examined the effects of water exercise [47, 54], which is also a part of other interventions [43, 45, 56, 59]. Although seven articles were included in the comparison, our analysis may suggest that exercise intervention alone does not allow for an increase in physical activity regardless of the type of exercise. Therefore, exercise alone might be insufficient to improve physical activity at either the endpoint or final follow-up in OA patients.

Likewise, we found very low evidence that educational intervention alone is ineffective for increasing physical activity levels at each time point. This result suggests that it may be difficult to improve physical activity using educational interventions alone in patients with hip or knee OA. With a chronic painful condition like OA, patients sometimes think that physical activity is harmful and ineffective for management of their conditions [61]. To counter this misconception, patient education has recently been recommended as a first-line management strategy as well as exercise therapy [60, 62, 63]. However, the patient education included in the studies we examined was not sufficient to increase physical activity, suggesting that patient education alone is not sufficient for behavioral modification. The effect varied from article to article, and the results differed depending on which article was excluded at follow up. In other words, the evidence regarding the effectiveness of patient education at follow-up is not yet consistent, and further RCTs are needed to examine effective interventions. For example, Shahine et al. [55] shows a strong effect at end point, but the type of intervention was not as specific as in other articles, except for intervention frequency. This may indicate that effect of patient education might be influenced by the frequency of feedback rather than the content of the education. Therefore, it seems important to note that high-frequency feedback may contribute to increased physical activity.

Although there was no effectiveness in exercise or educational intervention alone in increasing physical activity, combination therapy of exercise and educational intervention does appear to be effective for increasing physical activity at the end point, with the quality of the evidence rated as very low. Although the contents of the exercise and educational interventions of single and combination therapy were similar, no effectiveness was found in the single intervention of exercise or educational intervention, but it was found in combination therapy. Again, OA patients sometimes avoid physical activities because they believe that exercise will worsen their conditions [61]. However, accurate knowledge about the importance of physical activities facilitates adherence to the practice of exercise [64]. Based on these findings, educational intervention combined with exercise increases adherence to physical activity, which leads to behavioral modifications for managing OA symptoms. Recently, a multidisciplinary approach for OA patients has been advocated to improve their several symptoms [65]. Our current result that combination therapy for exercise and educational intervention is recommended for increasing physical activity may contribute to the importance of a multidisciplinary approach for OA patients. Meanwhile, there was not a difference physical activity levels at follow-up between combination therapy of exercise and educational intervention and pure control or placebo with the quality of the evidence rated as very low. This result may suggest that combination therapy of exercise and educational intervention cannot lead to behavioral modifications until follow up. Intervention strategies examined in previous studies have not reported increases in physical activity or maintenance of behavior change at follow-up, and further investigation of intervention strategies is needed. However, some papers reported increases in low-intensity physical activity and steps. Behavior modification affected by combination therapy for exercise and educational intervention might persist only for low physical activity levels and not for high-intensity physical activity levels. Therefore, controlled analysis of outcomes would be helpful in the development of future intervention strategies.

We also investigated the effects of exercise and/or educational intervention on osteoarthritis-associated pain. According to the results, exercise is effective for pain management. It is well known that exercise is effective for pain management in chronic pain conditions [16], which supports our results. The present study may provide evidence that exercise is effective for pain in patients with OA, despite its lack of effect on physical activity. Exercise may be another mechanism of decreasing pain; this mechanism is sometimes called exercise-induced hypoalgesia and is related to the exercise intensity [66, 67]. In addition, exercise was also effective for pain at follow-up, with low evidence. Therefore, as a point of pain management, the effects of exercise remain effective through follow up. This finding may suggest that regular exercise is effective for pain control and, persists even after the exercise intervention finishes.

The educational intervention was also effective for pain at the endpoint, but not at follow-up. Interestingly, these results of educational intervention for pain were similar to the findings of an article that investigated the effectiveness of pain neurophysiology education on musculoskeletal pain [68], which was also part of the education in a knee osteoarthritis study [25]. In other words, educational intervention alone might be effective for short-term effects. It might be that educational intervention modifies the way we think about pain while having no effect on the damaged tissue itself. Importantly, there was evidence, albeit low by the GRADE scale, that the combination therapy of exercise and educational intervention is effective for pain management both at the end point and at follow up. These results are similar to the article of Pitsillides et al. [69], although the control condition of that article is a mixture of the usual control and single interventions. The results of our current study is very important because the paper has a strict control group. In accordance with our results, combination therapy of exercise and educational intervention may have a long-term effect on pain management by affecting both the inflamed tissue and the perception of pain.

We determined the evidence quality using the GRADEpro guideline development tool, which consists of four components: risk of bias, inconsistency, indirectness, and imprecision. At the risk-of-bias component, almost all studies demonstrated high risk for overall risk of bias. Notably, bias of the randomization process and bias of measurement of the outcome are were very common in papers with a high risk of bias. Four of the five comparisons remained the same even when heterogeneous data were excluded, although one comparison (physical activity, educational intervention vs. control at follow-up) had different results when heterogeneous data were excluded. Additionally, we used the random-effects model for statistical analysis in this study. Therefore, it may not be necessary to consider the inconsistency component in each comparison, except for one comparison (physical activity, educational intervention, vs. control at follow-up) in this study. In the indirectness component, we chose “not serious” in all comparisons because there were no data where we needed to downgrade the results of indirectness according to previous guidelines [40]. In addition, we assigned “not serious” to most of the comparisons regarding data imprecision, except for one comparison (physical activity, educational intervention, vs. control at follow-up). This is because there were over 400 participants in each comparison, which previously described the criterion of imprecision data component [41]. In the publication bias component, we chose “undetected” in all comparisons because there were fewer articles included in each comparison. Therefore, we used the random-effects model to compare each outcome because publication bias was not considered. For these reasons, the comparisons in this study were assigned “low” to “very low” level of quality of evidence. If the risk of bias included in the study, especially the randomization process and dropouts in the participants, could be clarified and thus eliminated, the evidence level might be upgraded.

We found that a combination therapy of exercise and educational intervention improved physical activity and pain in patients with hip or knee OA at the end point. However, at the end of follow-up, this therapy did not increase the physical activity. Increased physical activity has been reported to inhibit OA [9], and it is known to improve physical function [9, 11]. According to the results and literature review, educational intervention combined with exercise upregulates exercise adherence [64], thereby increasing physical activity, which might promote the efficacy of exercise on pain. Although there was no effect on the physical activity at follow-up, we can recommend providing combination therapy of exercise and educational intervention to improve physical activity and pain with low evidence. Clearly, it is important to continue exercise, and patient education is essential for improving exercise adherence. However, further research is required to clarify the relationship between changes in physical activity and pain using a combination therapy of exercise and educational intervention.

This study had some limitations. First, we recruited all RCTs that investigated the effectiveness of exercise and/or educational intervention for patients with OA rather than specific exercise and educational content. In other words, the current study did not restrict either the methods and place of exercise and/or educational intervention, or the frequency and duration of these interventions. Thus, there is wide variation among the exercise and educational interventions applied in the studies, which may have affected the results. Additional studies are needed to clarify which type of intervention is most effective in increasing the physical activity in patients with knee or hip OA. Second, this study did not define the inclusion criteria for OA severity, and no subgroup analyses were performed according to OA severity. The effects of exercise and/or educational intervention may be affected by OA severity. Further studies are required to clarify this issue. Third, we did not use controlled vocabulary terms such as MeSH in the search strategy. If MeSH had been adopted, the number of articles identified might have been higher than the number found in this study. Fourth, we only searched the articles written in English, which might create a bias. Therefore, it is important to pursue studies written in other languages. Finally, we only investigated physical activity and pain in patients with OA. As mentioned above, there are several locomotive disabilities, such as muscle weakness and walking disability in hip/knee OA patients. Although there were differences in pain reduction, further studies are needed to clarify the effects of exercise and/or educational intervention for other locomotive disabilities.

## Conclusion

Our findings suggest that a combination therapy of exercise and educational intervention is more effective in increasing the amount of long-term physical activity and reducing pain in patients with knee or hip OA. Conversely, exercise or educational intervention alone is not effective at improving long-term physical activity. Improvements in long-term physical activity through the combination of exercise and educational intervention may secondarily improve pain. This meta-analysis provides evidence that combination therapy contributes to improvement of pain and physical activity in patients with OA. It is expected that further clinical studies will investigate both physical activity and pain in patients with OA.

## Supporting information

S1 File Search term and strategy of this study. (DOCX)

S2 File Individual data of RoB 2 in each outcome. (DOCX)

S3 File Reasons for exclusion in each RCT. (DOCX)

S4 File PRISMA 2020 checklist. (DOCX)

S5 File PRISMA 2020 for abstracts checklist. (DOCX)

## References

[1] Carmona-Terés V, Lumillo-Gutiérrez I, Jodar-Fernández L, Rodriguez-Blanco T, Moix-Queraltó J, Pujol-Ribera E, et al. Effectiveness and cost-effectiveness of a health coaching intervention to improve the lifestyle of patients with knee osteoarthritis: cluster randomized clinical trial. BMC Musculoskelet Disord. 2015; 16: 38. doi: 10.1186/s12891-015-0501-x 25887078 PMC4344994

[2] Lawrence RC, Felson DT, Helmick CG, Arnold LM, Choi H, Deyo RA, et al. Estimates of the prevalence of arthritis and other rheumatic condition in the United States. Part II. Arthritis Rheum 2008; 58: 26–35. 18163497 10.1002/art.23176 PMC3266664

[3] Aldosari AA, Majadah S, Amer KA, Alamri HH, Althomali RN, Alqahtani RF, et al. The Association Between Physical Activity Level and Severity of Knee Osteoarthritis: A Single Centre Study in Saudi Arabia. Cureus. 2022; 14: e24377. doi: 10.7759/cureus.24377 35611031 PMC9124548

[4] Bricca A, Wirth W, Juhl CB, Kemnitz J, Hunter DJ, Kwoh CK, et al. Moderate physical activity and prevention of cartilage loss in people with knee osteoarthritis: data from the osteoarthritis initiative. Arthritis Care Res 2019; 71: 218–226. doi: 10.1002/acr.23791 30339323

[5] Thoma LM, Dunlop D, Song J, Lee J, Tudor-Locke C, Aguiar EJ, et al. Are Older Adults With Symptomatic Knee Osteoarthritis Less Active Than the General Population? Analysis From the Osteoarthritis Initiative and the National Health and Nutrition Examination Survey. Arthritis Care Res (Hoboken). 2018; 70: 1448–1454. 29468844 10.1002/acr.23511 PMC6105574

[6] Dunlop DD, Song J, Semanik PA, Chang RW, Sharma L, Bathon JM, et al. Objective physical activity measurement in the osteoarthritis initiative: Are guidelines being met? Arthritis Rheum 2011; 63: 3372–3382. doi: 10.1002/art.30562 21792835 PMC3205278

[7] Hirata S, Ono R, Yamada M, Takikawa S, Nishiyama T, Hasuda K, et al. Ambulatory physical activity, disease severity, and employment status in adult women with osteoarthritis of the hip. J Rheumatol 2006; 33: 939–945. 16652424

[8] Song J, Chang AH, Chang RW, Lee J, Pinto D, Hawker G, et al. Relationship of knee pain to time in moderate and light physical activities: data from osteoarthritis initiative. Semin Arthritis Rheum 2018; 47: 683–688. doi: 10.1016/j.semarthrit.2017.10.005 29103557 PMC5866183

[9] Iijima H, Fukutani N, Isho T, Yamamoto Y, Hiraoka M, Miyanobu K, et al. Relationship between pedometer-based physical activity and physical function in patients with osteoarthritis of the knee: a cross-sectional study. Arch Phys Med Rehabil 2017; 98: 1382–1388. doi: 10.1016/j.apmr.2016.12.021 28131701

[10] Murphy SL, Niemiec SS, Lyden AK, Kratz AL. Pain, fatigue, and physical activity in osteoarthritis: the moderating effects of pain- and fatigue-related activity interference. Arch Phys Med Rehabil 2016; 97: 201–209. 10.1016/j.apmr.2015.05.025 27207435

[11] Dunlop DD, Song J, Semanik PA, Sharma L, Chang RW. Physical activity levels and functional performance in the osteoarthritis initiative: a graded relationship. Arthritis Rheum 2011; 63: 127–136. doi: 10.1002/art.27760 20862681 PMC3010474

[12] Stubbs B, Hurley M, Smith T. What are the factors that influence physical activity participation in adults with knee and hip osteoarthritis? A systematic review of physical activity correlates. Clin Rehabil. 2015; 29: 80–94. doi: 10.1177/0269215514538069 24917590

[13] Vlaeyen JWS, Linton SJ. Fear-avoidance and its consequences in chronic musculoskeletal pain: a state of the art. Pain 2000; 85: 317–332. doi: 10.1016/S0304-3959(99)00242-0 10781906

[14] Budarick AR, Moyer RF. Linking physical activity with clinical, functional, and structural outcomes: an evidence map using the Osteoarthritis Initiative. Clin Rheumatol. 2022; 41: 965–975. doi: 10.1007/s10067-021-05995-y 34802082

[15] Åkesson KS, Sundén A, Hansson EE, Stigmar K. Physiotherapists’ experiences of osteoarthritis guidelines in primary health care—an interview study. BMC Fam Pract. 2021; 22: 259. doi: 10.1186/s12875-021-01611-9 34969369 PMC8717645

[16] Geneen LJ, Moore RA, Clarke C, Martin D, Colvin LA, Smith BH. Physical activity and exercise for chronic pain in adults: an overview of Cochrane Reviews. Cochrane Database Syst Rev 2017; 4: CD011279. doi: 10.1002/14651858.CD011279.pub3 28436583 PMC5461882

[17] Fransen M, McConnell S, Harmer AR, Van der Esch M, Simic M, Bennell KL. Exercise for osteoarthritis of the knee. Cochrane Database Syst Rev. 2015; 1: CD004376. doi: 10.1002/14651858.CD004376.pub3 25569281 PMC10094004

[18] McAlindon TE, Bannuru RR, Sullivan MC, Arden NK, Berenbaum F, Bierma-Zeinstra SM, et al. OARSI guidelines for the non-surgical management of knee osteoarthritis. Osteoarthritis Cartilage 2014; 22: 363–388. doi: 10.1016/j.joca.2014.01.003 24462672

[19] Roos EM, Juhl CB. Osteoarthritis 2012 year in review: rehabilitation and outcomes. Osteoarthritis Cartilage 2012; 20: 1477–1483. doi: 10.1016/j.joca.2012.08.028 22960093

[20] Oliveira CB, Franco MR, Maher CG, Christine Lin CW, Morelhão PK, Araújo AC, et al. Physical activity interventions for increasing objectively measured physical activity levels in patients with chronic musculoskeletal pain: a systematic review. Arthritis Care Res 2016; 68: 1832–1842. doi: 10.1002/acr.22919 27111744

[21] Williamson W, Kluzek S, Roberts N, Richards J, Arden N, Leeson P, et al. Behavioural physical activity interventions in participants with lower-limb osteoarthritis: a systematic review with meta-analysis. BMJ Open 2015; 5: e007642. doi: 10.1136/bmjopen-2015-007642 26260348 PMC4538274

[22] Moseng T, Dagfinrud H, van Bodegom-Vos L, Dziedzic K, Hagen KB, Natvig B, et al. Low adherence to exercise may have influenced the proportion of OMERACT-OARSI responders in an integrated osteoarthritis care model: secondary analyses from a cluster-randomised stepped-wedge trial. BMC Musculoskelet Disord 2020; 21: 236. doi: 10.1186/s12891-020-03235-z 32284049 PMC7155273

[23] Schlenk EA, Fitzgerald GK, Rogers JC, Kwoh CK, Sereika SM. Promoting physical activity in older adults with knee osteoarthritis and hypertension: a randomized controlled trial. J Aging Phys Act 2020; 29: 207–218. doi: 10.1123/japa.2019-0498 32887850 PMC8450018

[24] Hughes SL, Seymour RB, Campbell RT, Huber G, Pollak N, Sharma L, et al. Long-term impact of Fit and Strong! on older adults with osteoarthritis. Gerontologist 2006; 46: 801–814. doi: 10.1093/geront/46.6.801 17169935

[25] Goff AJ, De Oliveira Silva D, Merolli M, Bell EC, Crossley KM, Barton CJ. Patient education improves pain and function in people with knee osteoarthritis with better effects when combined with exercise therapy: a systematic review. J Physiother 2021; 67: 177–189. doi: 10.1016/j.jphys.2021.06.011 34158270

[26] Geneen LJ, Martin DJ, Adams N, Clarke C, Dunbar M, Jones D, et al. Effect of education to facilitate knowledge about chronic pain for adults: A systematic review with meta-analysis. Syst Rev 2015; 4: 132. doi: 10.1186/s13643-015-0120-5 26428467 PMC4591560

[27] Moher D, Shamseer L, Clarke M, Ghersi D, Liberati A, Petticrew M, et al. Preferred reporting items for systematic review and meta-analysis protocols (PRISMA-P) 2015 statement. Syst Rev 2015; 4: 1. doi: 10.1186/2046-4053-4-1 25554246 PMC4320440

[28] Page MJ, Moher D, Bossuyt PM, Boutron I, Hoffmann TC, Mulrow CD, et al. PRISMA 2020 explanation and elaboration: updated guidance and exemplars for reporting systematic reviews. BMJ 2021; 372: n160. doi: 10.1136/bmj.n160 33781993 PMC8005925

[29] Higgins JPT, Thomas J, Chandler J, Cumpston M, Li T, Page MJ, et al. Cochrane Handbook for Systematic Reviews of Interventions. 2nd Edition. Chichester (UK): John Wiley & Sons, 2019.

[30] Higgins JPT, Thomas J, Chandler J, Cumpston M, Li T, Page MJ, et al. Cochrane Handbook for Systematic Reviews of Interventions version 6.3 (updated February 2022). Cochrane, 2022. Available from www.training.cochrane.org/handbook.

[31] Sterne JAC, Savović J, Page MJ, Elbers RG, Blencowe NS, Boutron I, et al. RoB 2: a revised tool for assessing risk of bias in randomised trials. BMJ. 2019; 366: l4898. doi: 10.1136/bmj.l4898 31462531

[32] Rethlefsen ML, Kirtley S, Waffenschmidt S, Ayala AP, Moher D, Page MJ, et al. PRISMA-S: an extension to the PRISMA Statement for Reporting Literature Searches in Systematic Reviews. Syst Rev. 2021; 10: 39. doi: 10.1186/s13643-020-01542-z 33499930 PMC7839230

[33] Abrams KR, Gillies CL, Lambert PC. Meta-analysis of heterogeneously reported trials assessing change from baseline. Stat Med 2005; 24: 3823–3844. doi: 10.1002/sim.2423 16320285

[34] Follmann D, Elliott P, Suh I, Cutler J. Variance imputation for overviews of clinical trials with continuous response. J Clin Epidemiol 1992; 45: 769–773. doi: 10.1016/0895-4356(92)90054-q 1619456

[35] Resende L, Merriwether E, Rampazo ÉP, Dailey D, Embree J, Deberg J, et al. Meta-analysis of transcutaneous electrical nerve stimulation for relief of spinal pain. Eur J Pain 2018; 22: 663–678. doi: 10.1002/ejp.1168 29282846

[36] Dantas LO, Moreira RFC, Norde FM, Mendes Silva Serrao PR, Alburquerque-Sendín F, Salvini TF. The effects of cryotherapy on pain and function in individuals with knee osteoarthritis: a systematic review of randomized controlled trials. Clin Rehabil 2019; 33: 1310–1319. doi: 10.1177/0269215519840406 30957514

[37] Balshem H, Helfand M, Schünemann HJ, Oxman AD, Kunz R, Brozek J. et al. GRADE guidelines: 3. Rating the quality of evidence. J Clin Epidemiol 2011; 64: 401–406. doi: 10.1016/j.jclinepi.2010.07.015 21208779

[38] Guyatt GH, Oxman AD, Vist G, Kunz R, Brozek J, Alonso-Coello P, et al. GRADE guidelines: 4. Rating the quality of evidence—study limitations (risk of bias). J Clin Epidemiol 2011; 64: 407–415. doi: 10.1016/j.jclinepi.2010.07.017 21247734

[39] Guyatt GH, Oxman AD, Kunz R, Woodcock J, Brozek J, Helfand M, et al. GRADE guidelines: 7. Rating the quality of evidence—inconsistency. J Clin Epidemiol 2011; 64: 1294–1302. doi: 10.1016/j.jclinepi.2011.03.017 21803546

[40] Guyatt GH, Oxman AD, Kunz R, Woodcock J, Brozek J, Helfand M. et al. GRADE guidelines: 8. Rating the quality of evidence—indirectness. J Clin Epidemiol 2011; 64: 1303–1310. doi: 10.1016/j.jclinepi.2011.04.014 21802903

[41] Guyatt GH, Oxman AD, Kunz R, Brozek J, Alonso-Coello P, Rind D, et al. GRADE guidelines 6. Rating the quality of evidence—imprecision. J Clin Epidemiol 2011; 64: 1283–1293. doi: 10.1016/j.jclinepi.2011.01.012 21839614

[42] Guyatt GH, Oxman AD, Montori V, Vist G, Kunz R, Brozek J, et al. GRADE guidelines: 5. Rating the quality of evidence—publication bias. J Clin Epidemiol 2011; 64: 1277–1282. doi: 10.1016/j.jclinepi.2011.01.011 21802904

[43] Allen KD, Arbeeva L, Callahan LF, Golightly YM, Goode AP, Heiderscheit BC, et al. Physical therapy vs internet-based exercise training for patients with knee osteoarthritis: results of a randomized controlled trial. Osteoarthritis Cartilage 2018; 26: 383–396. doi: 10.1016/j.joca.2017.12.008 29307722 PMC6021028

[44] Bartholdy C, Bliddal H, Henriksen M. Effectiveness of text messages for decreasing inactive behaviour in patients with knee osteoarthritis: a pilot randomised controlled study. Pilot Feasibility Stud 2019; 5:112. doi: 10.1186/s40814-019-0494-6 31516729 PMC6732192

[45] Bennell KL, Egerton T, Martin J, Abbott JH, Metcalf B, McManus F, et al. Effect of physical therapy on pain and function in patients with hip osteoarthritis: a randomized clinical trial. JAMA 2014; 311: 1987–1997. doi: 10.1001/jama.2014.4591 24846036

[46] Gilbert AL, Lee J, Ehrlich-Jones L, Semanik PA, Song J, Pellegrini CA, et al. A randomized trial of a motivational interviewing intervention to increase lifestyle physical activity and improve self-reported function in adults with arthritis. Semin Arthritis Rheum 2018; 47: 732–740. doi: 10.1016/j.semarthrit.2017.10.003 29096934 PMC5866185

[47] Hinman RS, Heywood SE, Day AR. Aquatic physical therapy for hip and knee osteoarthritis: results of a single-blind randomized controlled trial. Phys Ther 2007; 87: 32–43. doi: 10.2522/ptj.20060006 17142642

[48] Hinman RS, Campbell PK, Lawford BJ, Briggs AM, Gale J, Bills C, et al. Does telephone-delivered exercise advice and support by physiotherapists improve pain and/or function in people with knee osteoarthritis? Telecare randomised controlled trial. Br J Sports Med 2020; 54: 790–797. doi: 10.1136/bjsports-2019-101183 31748198

[49] Hughes SL, Seymour RB, Campbell R, Pollak N, Huber G, Sharma L. Impact of the fit and strong intervention on older adults with osteoarthritis. Gerontologist 2004; 44: 217–228. doi: 10.1093/geront/44.2.217 15075418

[50] Li LC, Sayre EC, Xie H, Clayton C, Feehan LM. A community-based physical activity counselling program for people with knee osteoarthritis: feasibility and preliminary efficacy of the Track-OA study. JMIR Mhealth Uhealth 2017; 5: e86. doi: 10.2196/mhealth.7863 28652228 PMC5504340

[51] Li LC, Sayre EC, Xie H, Falck RS, Best JR, Liu-Ambrose T, et al. Efficacy of a community-based technology-enabled physical activity counseling program for people with knee osteoarthritis: proof-of-concept study. J Med Internet Res 2018; 20: e159. doi: 10.2196/jmir.8514 29712630 PMC5952118

[52] Li LC, Feehan LM, Xie H, Lu N, Shaw CD, Gromala D, et al. Effects of a 12-week multifaceted wearable-based program for people with knee osteoarthritis: randomized controlled trial. JMIR Mhealth Uhealth 2020; 8: e19116. doi: 10.2196/19116 32618578 PMC7367519

[53] Murphy SL, Janevic MR, Lee P, Williams DA. Occupational therapist-delivered cognitive-behavioral therapy for knee osteoarthritis: a randomized pilot study. Am J Occup Ther 2018; 72: 7205205040p1–7205205040p9. doi: 10.5014/ajot.2018.027870 30157016 PMC6114193

[54] Rewald S, Lenssen AFT, Emans PJ, de Bie RA, van Breukelen G, Mesters I. Aquatic cycling improves knee pain and physical functioning in patients with knee osteoarthritis: a randomized controlled trial. Arch Phys Med Rehabil 2020; 101: 1288–1295. doi: 10.1016/j.apmr.2019.12.023 32169459

[55] Shahine NF, El Ashri NI, Senna MK, Abd Elhameed SH. Effect of a pedometer based aerobic walking program on pain and function among elderly patients with knee osteoarthritis. EJMCM 2020; 7: 790–799.

[56] Vincent KR, Vincent HK. Concentric and eccentric resistance training comparison on physical function and functional pain outcomes in knee osteoarthritis: a randomized controlled trial. Am J Phys Med Rehabil 2020; 99: 932–940. doi: 10.1097/PHM.0000000000001450 32324615

[57] Waller B, Munukka M, Rantalainen T, Lammentausta E, Nieminen MT, Kiviranta I, et al. Effects of high intensity resistance aquatic training on body composition and walking speed in women with mild knee osteoarthritis: a 4-month RCT with 12-month follow-up. Osteoarthritis Cartilage 2017; 25: 1238–1246. doi: 10.1016/j.joca.2017.02.800 28263901

[58] Wallis JA, Webster KE, Levinger P, Singh PJ, Fong C, Taylor NF. A walking program for people with severe knee osteoarthritis did not reduce pain but may have benefits for cardiovascular health: a phase II randomised controlled trial. Osteoarthritis Cartilage 2017; 25: 1969–1979. doi: 10.1016/j.joca.2016.12.017 28011099

[59] Wortley M, Zhang S, Paquette M, Byrd E, Baumgartner L, Klipple G, et al. Effects of resistance and Tai Ji training on mobility and symptoms in knee osteoarthritis patients. JSHS 2013; 2: 209–214.

[60] Bannuru RR, Osani MC, Vaysbrot EE, Arden NK, Bennell K, Bierma-Zeinstra SMA, et al. OARSI guidelines for the non-surgical management of knee, hip, and polyarticular osteoarthritis. Osteoarthritis Cartilage. 2019; 27: 1578–1589. doi: 10.1016/j.joca.2019.06.011 31278997

[61] Goldchmit SM, de Queiroz MC, Dos Anjos Rabelo ND, Junior WR, Polesello GC. Patient Education in Orthopedics: the Role of Information Design and User Experience. Curr Rev Musculoskelet Med. 2021; 14: 9–15. doi: 10.1007/s12178-020-09683-3 33403625 PMC7930126

[62] Hunter DJ, Bierma-Zeinstra S. Osteoarthritis. Lancet. 2019; 393: 1745–1759. doi: 10.1016/S0140-6736(19)30417-9 31034380

[63] Kolasinski SL, Neogi T, Hochberg MC, Oatis C, Guyatt G, Block J, et al. 2019 American College of Rheumatology/Arthritis Foundation Guideline for the Management of Osteoarthritis of the Hand, Hip, and Knee. Arthritis Rheumatol. 2020; 72: 220–233. doi: 10.1002/art.41142 31908163 PMC10518852

[64] Kanavaki AM, Rushton A, Efstathiou N, Alrushud A, Klocke R, Abhishek A, et al. Barriers and facilitators of physical activity in knee and hip osteoarthritis: a systematic review of qualitative evidence. BMJ Open. 2017; 7: e017042. doi: 10.1136/bmjopen-2017-017042 29282257 PMC5770915

[65] Iolascon G, Ruggiero C, Fiore P, Mauro GL, Moretti B, Tarantino U. Multidisciplinary integrated approach for older adults with symptomatic osteoarthritis: SIMFER and SI-GUIDA Joint Position Statement. Eur J Phys Rehabil Med. 2020; 56: 112–119. doi: 10.23736/S1973-9087.19.05837-4 31742367

[66] Rice D, Nijs J, Kosek E, Wideman T, Hasenbring MI, Koltyn K, et al. Exercise-induced hypoalgesia in pain-free and chronic pain populations: state of the art and future directions. J Pain 2019; 20: 1249–1266. doi: 10.1016/j.jpain.2019.03.005 30904519

[67] Wewege MA, Jones MD. Exercise-induced hypoalgesia in healthy individuals and people with chronic musculoskeletal pain: a systematic review and meta-analysis. J Pain 2021; 22: 21–31. doi: 10.1016/j.jpain.2020.04.003 32599154

[68] Bülow K, Lindberg K, Vaegter HB, Juhl CB. Effectiveness of Pain Neurophysiology Education on Musculoskeletal Pain: A Systematic Review and Meta-Analysis. Pain Med. 2021; 22: 891–904. doi: 10.1093/pm/pnaa484 33764394

[69] Pitsillides A, Stasinopoulos D, Giannakou K. The effects of cognitive behavioural therapy delivered by physical therapists in knee osteoarthritis pain: A systematic review and meta-analysis of randomized controlled trials. J Bodyw Mov Ther. 2021; 25: 157–164. doi: 10.1016/j.jbmt.2020.11.002 33714488

