# Supplementary material for: Effect of exercise and/or educational interventions on physical activity and pain in patients with hip/knee osteoarthritis: A systematic review with meta-analysis
Source: PLoS One. 2022 Nov 21;17(11):e0275591. doi: 10.1371/journal.pone.0275591 (PMC9678259; doi:10.1371/journal.pone.0275591)
Supplement: S1 File — (DOCX) [file pone.0275591.s001.docx]

Search term for this study

Population

“osteoarthritis”

Intervention (exercise)

“exercise,” “aerobic,” “resistance,” “isometric,” “stabilization,” “tai chi,” “yoga,” “Pilates,” “qigong,” “training,” “rehabilitation,”

Intervention (educational intervention)

“lecture,” “cognitive behavioral therapy,” “behavioral medicine approach,”

“behavior management,” “pacing,” “lifestyle management,” “coping,” “education,”

Outcome (physical activity)

“physical activity,” “activity,” “step,” “steps,” “METs,” “sedentary,” “moderate,” “vigorous,”

“vector magnitude,” “MVPA,” “pedometer,” “accelerometer”

Outcome (pain)

“pain,” “VAS,” “visual analog scale,” “NRS,” “numeral rating scale,” “WOMAC,”

“Western Ontario and McMaster Universities Osteoarthritis Index”

Article type

“RCT,” “randomized controlled trial”

MEDLINE (PubMed)

Search Period: earliest records to April 30, 2022

Search strategy

| # | Searches | Search fields | Results |
| --- | --- | --- | --- |
| 1 | RCT | All fields | 30,453 |
| 2 | randomized controlled trial | All fields | 757,011 |
| 3 | #1 or #2 | All fields | 767,144 |
| 4 | osteoarthritis | All fields | 104,375 |
| 5 | exercise | All fields | 531,119 |
| 6 | aerobic | All fields | 571,838 |
| 7 | resistance | All fields | 1,309,640 |
| 8 | isometric | All fields | 527,658 |
| 9 | stabilization | All fields | 827,198 |
| 10 | tai chi | All fields | 3,441 |
| 11 | yoga | All fields | 6,899 |
| 12 | Pilates | All fields | 789 |
| 13 | qigong | All fields | 974 |
| 14 | training | All fields | 2,283,488 |
| 15 | rehabilitation | All fields | 727,877 |
| 16 | #5 or #6 or #7 or #8 or #9 or #10 or #11 or #12 or #13 or #14 or #15 | All fields | 5,156,289 |
| 17 | lecture | All fields | 33,975 |
| 18 | CBT | All fields | 13,866 |
| 19 | cognitive behavioral therapy | All fields | 52,872 |
| 20 | behavioral medicine approach | All fields | 18,511 |
| 21 | behavior management | All fields | 675,747 |
| 22 | pacing | All fields | 63,134 |
| 23 | lifestyle management | All fields | 46,841 |
| 24 | coping | All fields | 181,583 |
| 25 | education | All fields | 2,078,181 |
| 26 | #17 or #18 or #19 or #20 or #21 or #22 or #23 or #24 or #25 | All fields | 2,861,232 |
| 27 | physical activity | All fields | 642,867 |
| 28 | activity | All fields | 5,751,919 |
| 29 | step | All fields | 473,740 |
| 30 | steps | All fields | 233,432 |
| 31 | METs | All fields | 14,162 |
| 32 | sedentary | All fields | 41,056 |
| 33 | moderate | All fields | 672,932 |
| 34 | vigorous | All fields | 28,838 |
| 35 | vector magnitude | All fields | 5,589 |
| 36 | MVPA | All fields | 5,671 |
| 37 | pedometer | All fields | 2,950 |
| 38 | accelerometer | All fields | 17,273 |
| 39 | #27 or #28 or #29 or #30 or #31 or #32 or #33 or #34 or #35 or #36 or #37 or #38 | All fields | 6,933,254 |
| 40 | pain | All fields | 945,918 |
| 41 | VAS | All fields | 61,915 |
| 42 | visual analog scale | All fields | 63,266 |
| 43 | NRS | All fields | 14,399 |
| 44 | numeral rating scale | All fields | 33 |
| 45 | WOMAC | All fields | 5,512 |
| 46 | Western Ontario and McMaster Universities Osteoarthritis Index | All fields | 4,181 |
| 47 | #40 or #41 or #42 or #43 or #44 or #45 or #46 | All fields | 995,883 |
| 48 | #3 and #4 and #16 and #39 and #47 | All fields | 1,830 |
| 49 | #3 and #4 and #26 and #39 and #47 | All fields | 656 |

PEDro

Search Period: earliest records to April 30, 2022

Search strategy

| # | Abstract & Title | Therapy | Problem | Body Part | Method | Results |
| --- | --- | --- | --- | --- | --- | --- |
| 1 | osteoarthritis | behaviour modification | pain | thigh or hip | clinical trial | 29 |
| 2 | osteoarthritis | education | pain | thigh or hip | clinical trial | 62 |
| 3 | osteoarthritis | fitness training | pain | thigh or hip | clinical trial | 32 |
| 4 | osteoarthritis | health promotion | pain | thigh or hip | clinical trial | 1 |
| 5 | osteoarthritis | strength training | pain | thigh or hip | clinical trial | 112 |
| 6 | osteoarthritis | behaviour modification | pain | lower leg or knee | clinical trial | 102 |
| 7 | osteoarthritis | education | pain | lower leg or knee | clinical trial | 196 |
| 8 | osteoarthritis | fitness training | pain | lower leg or knee | clinical trial | 211 |
| 9 | osteoarthritis | health promotion | pain | lower leg or knee | clinical trial | 13 |
| 10 | osteoarthritis | strength training | pain | lower leg or knee | clinical trial | 488 |

Scopus (Elsevier)

Published to April 30, 2022

Search strategy

| # | Search documents | Search whithin | Results |
| --- | --- | --- | --- |
| 1 | RCT | Article title, Abstract, Keywords | 72,860 |
| 2 | randomized controlled trial | Article title, Abstract, Keywords | 905,179 |
| 3 | #1 or #2 | Article title, Abstract, Keywords | 919,513 |
| 4 | osteoarthritis | Article title, Abstract, Keywords | 145,033 |
| 5 | exercise | Article title, Abstract, Keywords | 720,292 |
| 6 | aerobic | Article title, Abstract, Keywords | 180,843 |
| 7 | resistance | Article title, Abstract, Keywords | 2,365,763 |
| 8 | isometric | Article title, Abstract, Keywords | 63,886 |
| 9 | stabilization | Article title, Abstract, Keywords | 345,339 |
| 10 | tai chi | Article title, Abstract, Keywords | 3,897 |
| 11 | yoga | Article title, Abstract, Keywords | 12,299 |
| 12 | Pilates | Article title, Abstract, Keywords | 1,209 |
| 13 | qigong | Article title, Abstract, Keywords | 1,635 |
| 14 | training | Article title, Abstract, Keywords | 1,380,983 |
| 15 | rehabilitation | Article title, Abstract, Keywords | 393,193 |
| 16 | #5 or #6 or #7 or #8 or #9 or #10 or #11 or #12 or #13 or #14 or #15 | Article title, Abstract, Keywords | 5,071,454 |
| 17 | lecture | Article title, Abstract, Keywords | 108,939 |
| 18 | CBT | Article title, Abstract, Keywords | 20,916 |
| 19 | cognitive behavioral therapy | Article title, Abstract, Keywords | 35,320 |
| 20 | behavioral medicine approach | Article title, Abstract, Keywords | 6,369 |
| 21 | behavior management | Article title, Abstract, Keywords | 326,218 |
| 22 | pacing | Article title, Abstract, Keywords | 57,975 |
| 23 | lifestyle management | Article title, Abstract, Keywords | 40,389 |
| 24 | coping | Article title, Abstract, Keywords | 157,255 |
| 25 | education | Article title, Abstract, Keywords | 2,333,255 |
| 26 | #17 or #18 or #19 or #20 or #21 or #22 or #23 or #24 or #25 | Article title, Abstract, Keywords | 2,645,060 |
| 27 | physical activity | Article title, Abstract, Keywords | 523,860 |
| 28 | activity | Article title, Abstract, Keywords | 6,971,558 |
| 29 | step | Article title, Abstract, Keywords | 2,121,485 |
| 30 | steps | Article title, Abstract, Keywords | 2,121,485 |
| 31 | METs | Article title, Abstract, Keywords | 15,292 |
| 32 | sedentary | Article title, Abstract, Keywords | 58,071 |
| 33 | moderate | Article title, Abstract, Keywords | 927,177 |
| 34 | vigorous | Article title, Abstract, Keywords | 54,619 |
| 35 | vector magnitude | Article title, Abstract, Keywords | 27,006 |
| 36 | MVPA | Article title, Abstract, Keywords | 6,241 |
| 37 | pedometer | Article title, Abstract, Keywords | 4,198 |
| 38 | accelerometer | Article title, Abstract, Keywords | 64,534 |
| 39 | #27 or #28 or #29 or #30 or #31 or #32 or #33 or #34 or #35 or #36 or #37 or #38 | Article title, Abstract, Keywords | 9,728,628 |
| 40 | pain | Article title, Abstract, Keywords | 1,308,209 |
| 41 | VAS | Article title, Abstract, Keywords | 128,937 |
| 42 | visual analog scale | Article title, Abstract, Keywords | 110,759 |
| 43 | NRS | Article title, Abstract, Keywords | 15,916 |
| 44 | numeral rating scale | Article title, Abstract, Keywords | 53 |
| 45 | WOMAC | Article title, Abstract, Keywords | 6,139 |
| 46 | Western Ontario and McMaster Universities Osteoarthritis Index | Article title, Abstract, Keywords | 6,388 |
| 47 | #40 or #41 or #42 or #43 or #44 or #45 or #46 | Article title, Abstract, Keywords | 1,432,546 |
| 48 | #3 and #4 and #16 and #39 and #47 | Article title, Abstract, Keywords | 946 |
| 49 | #3 and #4 and #26 and #39 and #47 | Article title, Abstract, Keywords | 287 |

ProQuest

Selected databases: 6

Applied filters

Publication date: Before April 30 2022

Limit to: Peer reviewed

Search strategy

| # | Search terms | Search fields | Results |
| --- | --- | --- | --- |
| 1 | RCT | Anywhere | 120,461 |
| 2 | randomized controlled trial | Anywhere | 1,035,620 |
| 3 | #1 or #2 | Anywhere | 1,060,849 |
| 4 | osteoarthritis | Anywhere | 172,986 |
| 5 | exercise | Anywhere | 1,049,489 |
| 6 | aerobic | Anywhere | 296,498 |
| 7 | resistance | Anywhere | 3,439,731 |
| 8 | isometric | Anywhere | 83,317 |
| 9 | stabilization | Anywhere | 464,118 |
| 10 | tai chi | Anywhere | 30,442 |
| 11 | yoga | Anywhere | 28,286 |
| 12 | Pilates | Anywhere | 4,283 |
| 13 | qigong | Anywhere | 3,140 |
| 14 | training | Anywhere | 1,788,276 |
| 15 | rehabilitation | Anywhere | 616,260 |
| 16 | #5 or #6 or #7 or #8 or #9 or #10 or #11 or #12 or #13 or #14 or #15 | Anywhere | 5,295,148 |
| 17 | lecture | Anywhere | 331,371 |
| 18 | CBT | Anywhere | 43,031 |
| 19 | cognitive behavioral therapy | Anywhere | 254,047 |
| 20 | behavioral medicine approach | Anywhere | 373,221 |
| 21 | behavior management | Anywhere | 1,178,378 |
| 22 | pacing | Anywhere | 93,847 |
| 23 | lifestyle management | Anywhere | 249,443 |
| 24 | coping | Anywhere | 313,175 |
| 25 | education | Anywhere | 2,727,274 |
| 26 | #17 or #18 or #19 or #20 or #21 or #22 or #23 or #24 or #25 | Anywhere | 3,977,171 |
| 27 | physical activity | Anywhere | 1,721,968 |
| 28 | activity | Anywhere | 7,132,501 |
| 29 | step | Anywhere | 3,286,080 |
| 30 | steps | Anywhere | 3,286,080 |
| 31 | METs | Anywhere | 32,834 |
| 32 | sedentary | Anywhere | 123,698 |
| 33 | moderate | Anywhere | 1,811,955 |
| 34 | vigorous | Anywhere | 167,396 |
| 35 | vector magnitude | Anywhere | 235,154 |
| 36 | MVPA | Anywhere | 10,260 |
| 37 | pedometer | Anywhere | 11,465 |
| 38 | accelerometer | Anywhere | 54,973 |
| 39 | #27 or #28 or #29 or #30 or #31 or #32 or #33 or #34 or #35 or #36 or #37 or #38 | Anywhere | 9,473,850 |
| 40 | pain | Anywhere | 1,553,643 |
| 41 | VAS | Anywhere | 153,338 |
| 42 | visual analog scale | Anywhere | 83,035 |
| 43 | NRS | Anywhere | 30,070 |
| 44 | numeral rating scale | Anywhere | 1,674 |
| 45 | WOMAC | Anywhere | 10,162 |
| 46 | Western Ontario and McMaster Universities Osteoarthritis Index | Anywhere | 7,382 |
| 47 | #40 or #41 or #42 or #43 or #44 or #45 or #46 | Anywhere | 1,675,578 |
| 48 | #3 and #4 and #16 and #39 and #47 | Anywhere | 19,746 |
| 49 | #3 and #4 and #26 and #39 and #47 | Anywhere | 14,711 |
