# Supplementary material for: Effect of exercise and/or educational interventions on physical activity and pain in patients with hip/knee osteoarthritis: A systematic review with meta-analysis
Source: PLoS One. 2022 Nov 21;17(11):e0275591. doi: 10.1371/journal.pone.0275591 (PMC9678259; doi:10.1371/journal.pone.0275591)
Supplement: S2 File — (DOCX) [file pone.0275591.s002.docx]

1. RoB 2 on Physical Activity

1. RoB 2 on Pain

S2 Appendix. Individual data of RoB 2 in each outcome. Upper one shows RoB 2 on physical activity (A), and under one shows RoB 2 on pain (B). D1: bias arising from the randomization process, D2: bias due to deviations from intended interventions, D3: bias due to missing outcome data, D4: bias in measurement of the outcome, D5: bias in selection of the reported result.
