# Supplementary material for: Effect of exercise and/or educational interventions on physical activity and pain in patients with hip/knee osteoarthritis: A systematic review with meta-analysis
Source: PLoS One. 2022 Nov 21;17(11):e0275591. doi: 10.1371/journal.pone.0275591 (PMC9678259; doi:10.1371/journal.pone.0275591)
Supplement: S3 File — (DOCX) [file pone.0275591.s003.docx]

S3 Appendix. Reasons for exclusion

| **Study** | **Decision in manuscript** | **Additional notes** |
| --- | --- | --- |
| Abbott JH 2013 | Not evaluated physical activity or pain | Not listed the data on physical activity |
| Abbott JH 2019 | Not evaluated physical activity or pain | Not listed the data on physical activity |
| Adel J 2019 | Not a pure control group | Control group received exercise intervention. |
| Ahmed AF 2011 | Not evaluated physical activity or pain | Not listed the data on physical activity |
| Ahn YH 2020 | Not evaluated physical activity or pain | Not listed the data on physical activity |
| Alasfour M 2020 | Not a pure control group | Control group received both exercise and educational intervention. |
| Alfieri FM 2020 | Not evaluated physical activity or pain | Not listed the data on physical activity |
| Alghadir AH 2019 | Not evaluated physical activity or pain | Not listed the data on physical activity |
| Alkatan M 2016a | Not a pure control group | Control group received exercise intervention. |
| Alkatan M 2016b | Not a pure control group | Comparison of swimming and cycling exercise intervention. |
| Allen KD 2016 | Not a pure control group | Control group received exercise intervention. |
| Allen KD 2017 | Not a pure control group | Some patients in control group received injection. |
| Allen KD 2021 | Not a pure control group | Control group received educational intervention. |
| Altaş EU 2020 | Not a relevant intervention | Interventions include physical therapy (e.g., TENS). |
| An J 2021 | Not evaluated physical activity or pain | Not listed the data on physical activity |
| Anderson AM 2021 | Not evaluated physical activity or pain | Not listed the data on physical activity |
| Andersson ML 2006 | Not evaluated physical activity or pain | Not listed the data on physical activity |
| Anwer S 2014 | Not evaluated physical activity or pain | Not listed the data on physical activity |
| Arfaei Chitkar SS 2021 | Not evaluated physical activity or pain | Not listed the data on physical activity |
| Ashok C 2012 | Not evaluated physical activity or pain | Not listed the data on physical activity |
| Assar S 2020 | Not evaluated physical activity or pain | Not listed the data on physical activity |
| Aunger JA 2020 | Not evaluated physical activity or pain | Not listed the data on pain |
| Ay S 2013 | Not evaluated physical activity or pain | Not listed the data on physical activity |
| Azizi S 2020 | Not evaluated physical activity or pain | Not listed the data on physical activity |
| Azma K 2018 | Not evaluated physical activity or pain | Not listed the data on physical activity |
| Baker K 2020 | Several diseases included | Participants who underwent total knee arthroplasty |
| Baker KR 2001 | Not evaluated physical activity or pain | Not listed the data on physical activity |
| Bandak E 2019 | Not evaluated physical activity or pain | Not listed the data on physical activity |
| Barlow JH 2000 | Not evaluated physical activity or pain | Not listed the data on physical activity |
| Batterham SI 2011 | Not evaluated physical activity or pain | Not listed the data on physical activity |
| Bautch JC 2000 | Not evaluated physical activity or pain | Not listed the data on physical activity |
| Beaupre LA 2004 | Not evaluated physical activity or pain | Not listed the data on physical activity |
| Beckwée D 2017 | Not evaluated physical activity or pain | Not listed the data on physical activity |
| Bennell KL 2005 | Not evaluated physical activity or pain | Not listed the data on physical activity |
| Bennell KL 2011 | Wrong study type | Protocol study |
| Bennell KL 2012 | Wrong study type | Review article which abstract cannot be found in the primary search |
| Bennell KL 2014b | Not evaluated physical activity or pain | Not listed the data on physical activity |
| Bennell KL 2014c | Not evaluated physical activity or pain | Not listed the data on physical activity |
| Bennell KL 2015 | Not evaluated physical activity or pain | Not listed the data on physical activity |
| Bennell KL 2016 | Not a pure control group | Control group received either exercise or pain coping skill training. |
| Bennell KL 2017a | Not a pure control group | Control group received educational intervention and home-based exercise. |
| Bennell KL 2017b | Not evaluated physical activity or pain | Not listed the data on physical activity |
| Bennell KL 2018 | Not a pure control group | Control group received online education. |
| Bennell KL 2020 | Not a pure control group | Contlol group received two types of exercise interventions |
| Bennell KL 2022 | Not a relevant intervention | Intervention group received dietary intervention including consultations for dietitian |
| Berge DJ 2004 | Not evaluated physical activity or pain | Not listed the data on physical activity |
| Beselga C 2016 | Not evaluated physical activity or pain | Not listed the data on physical activity |
| Bieler T 2017 | Not a pure control group | Control group performed home-based exercise. |
| Bieler T 2021 | Not a pure control group | Control group received exercise intervention. |
| Birch S 2020 | Not evaluated physical activity or pain | Not listed the data on physical activity |
| Blackman F 2014 | Not evaluated physical activity or pain | Not listed the data on physical activity |
| Blixen CE 2004 | Not evaluated physical activity or pain | Not listed the data on physical activity |
| Bokaeian HR 2021 | Not a pure control group | Control group received exercise intervention. |
| Börjesson M 1996 | Not evaluated physical activity or pain | Not listed the data on physical activity |
| Bossen D 2013 | Not a pure control group | Control group received a letter about the information of physical activity and osteoarthritis. |
| Bove AM 2018 | Not evaluated physical activity or pain | Not listed the data on physical activity |
| Braghin RMB 2018 | Not evaluated physical activity or pain | Not listed the data on physical activity |
| Brantingham JW 2012 | Not evaluated physical activity or pain | Not listed the data on physical activity |
| Brismée JM 2007 | Not evaluated physical activity or pain | Not listed the data on physical activity |
| Broderick JE 2014 | Not evaluated physical activity or pain | Not listed the data on physical activity |
| Brosseau L 2012 | No data available on physical activity or pain | Leisure time activities and Other domestic activities were not appropriate for analysis. |
| Brown K 2014 | Not evaluated physical activity or pain | Not listed the data on physical activity |
| Bruce-Brand RA 2012 | Not evaluated physical activity or pain | Not listed the data on physical activity |
| Bryk FF 2016 | Not a pure control group | Control group received exercise intervention. |
| Bunsanong T 2021 | Not evaluated physical activity or pain | Not listed the data on physical activity |
| Burrows NJ 2014 | Not evaluated physical activity or pain | Not listed the data on physical activity |
| Buszewicz M 2006 | Not evaluated physical activity or pain | Not listed the data on physical activity |
| Çakir T 2016 | Not evaluated physical activity or pain | Not listed the data on physical activity |
| Calatayud J 2017 | Not evaluated physical activity or pain | Not listed the data on physical activity |
| Callaghan MJ 1995 | Not evaluated physical activity or pain | Not listed the data on physical activity |
| Carmona-Terés V 2015 | Not evaluated physical activity or pain | Not listed the data on physical activity |
| Casilda-López J 2017 | Not evaluated physical activity or pain | Not listed the data on physical activity |
| Chang TF 2012 | Not evaluated physical activity or pain | Not listed the data on physical activity |
| Chao J 2020 | Not a pure control group | Control group received analgesics. |
| Chassany O 2006 | Not evaluated physical activity or pain | Not listed the data on physical activity |
| Cheawthamai K 2014 | Not evaluated physical activity or pain | Not listed the data on physical activity |
| Chen H 2020 | Not evaluated physical activity or pain | Not listed the data on physical activity |
| Chen SM 2019 | Not a pure control group | Control group received exercise intervention. |
| Cheragh-Birjandi S 2020 | Not evaluated physical activity or pain | Not listed the data on physical activity |
| Cheung C 2014 | Not evaluated physical activity or pain | Not listed the data on physical activity |
| Cheung C 2017 | Not evaluated physical activity or pain | Not listed the data on physical activity |
| Cheung RTH 2018 | Not evaluated physical activity or pain | Not listed the data on physical activity |
| Cho Y 2015 | Not evaluated physical activity or pain | Not listed the data on physical activity |
| Choi YL 2015 | Not evaluated physical activity or pain | Not listed the data on physical activity |
| Chopp-Hurley JN 2017 | Not evaluated physical activity or pain | Not listed the data on physical activity |
| Christensen R 2015 | Not evaluated physical activity or pain | Not listed the data on physical activity |
| Claassen AAOM 2020 | Not evaluated physical activity or pain | Not listed the data on physical activity |
| Clarke SP 2017 | Not evaluated physical activity or pain | Not listed the data on physical activity |
| Coleman S 2012 | Not evaluated physical activity or pain | Not listed the data on physical activity |
| da Silva FS 2015 | Not evaluated physical activity or pain | Not listed the data on physical activity |
| das Nair R 2018 | Not evaluated physical activity or pain | Not listed the data on physical activity |
| Daskapan A 2013 | Not evaluated physical activity or pain | Not listed the data on physical activity |
| Davis HC 2019 | Not evaluated physical activity or pain | Not listed the data on physical activity |
| de Almeida AC 2020 | Not evaluated physical activity or pain | Not listed the data on physical activity |
| de Almeida AC 2021 | Not evaluated physical activity or pain | Not listed the data on physical activity |
| de Jong OR 2004 | Not evaluated physical activity or pain | Not listed the data on physical activity |
| de Oliveira 2012 | Not evaluated physical activity or pain | Not listed the data on physical activity |
| de Rezende MU 2016 | Not evaluated physical activity or pain | Not listed the data on physical activity |
| de Rezende MU 2017 | Not evaluated physical activity or pain | Not listed the data on physical activity |
| de Rooij M 2017 | No data available on physical activity or pain | Results of physical activity are not described in detail. |
| de Vos BC 2014 | Not evaluated physical activity or pain | Not listed the data on physical activity |
| de Zwart AH 2022 | Not a pure control group | Control group received exercise intervention. |
| DeVita P 2018 | Not evaluated physical activity or pain | Not listed the data on physical activity |
| Deyle GD 2000 | Not evaluated physical activity or pain | Not listed the data on physical activity |
| Deyle GD 2005 | Not evaluated physical activity or pain | Not listed the data on physical activity |
| Dharmasri CJ 2020 | Not evaluated physical activity or pain | Not listed the data on physical activity |
| Dias JM 2017 | Not evaluated physical activity or pain | Not listed the data on physical activity |
| Dias RC 2003 | Not evaluated physical activity or pain | Not listed the data on physical activity |
| Diracoglu D 2005 | Not evaluated physical activity or pain | Not listed the data on physical activity |
| D'Lima DD 1996 | Not evaluated physical activity or pain | Not listed the data on physical activity |
| Doiron-Cadrin P 2020 | Not evaluated physical activity or pain | Not listed the data on physical activity |
| Duarte N 2020 | No data available on physical activity or pain | Data are reported as median |
| Durst J 2020 | Not evaluated physical activity or pain | Not listed the data on physical activity |
| Dziedzic KS 2018 | Not evaluated physical activity or pain | Not listed the data on physical activity |
| Ebnezar J 2011 | Not evaluated physical activity or pain | Not listed the data on physical activity |
| Ebnezar J 2012a | Not evaluated physical activity or pain | Not listed the data on physical activity |
| Ebnezar J 2012b | Not evaluated physical activity or pain | Not listed the data on physical activity |
| Ebnezar J 2012c | Not evaluated physical activity or pain | Not listed the data on physical activity |
| Egerton T 2022 | Not a pure control group | Control group received educational intervention. |
| Ellegaard M 2020 | Not evaluated physical activity or pain | Not listed the data on physical activity |
| Escalante Y 2011 | Wrong study type | Systematic review |
| Eschalier B 2017 | Not evaluated physical activity or pain | Not listed the data on physical activity |
| Estébanez-de-Miguel E 2019 | Not evaluated physical activity or pain | Not listed the data on physical activity |
| Ettinger WH Jr 1997 | Not evaluated physical activity or pain | Not listed the data on physical activity |
| Evcik D 2002 | Not evaluated physical activity or pain | Not listed the data on physical activity |
| Falck RS 2018 | Not evaluated physical activity or pain | Not listed the data on physical activity |
| Farr JN 2010 | Not a pure control group | Control group received either exercise or educational intervention. |
| Fazaa A 2014 | Not evaluated physical activity or pain | Not listed the data on physical activity |
| Fernandes L 2017 | Not evaluated physical activity or pain | Not listed the data on physical activity |
| Fioravanti A 2015 | Not evaluated physical activity or pain | Not listed the data on physical activity |
| Fisken AL 2015 | Not a pure control group | Control group received exercise intervention. |
| Fitzgerald GK 2011 | Not evaluated physical activity or pain | Not listed the data on physical activity |
| Fitzgerald GK 2016 | Not evaluated physical activity or pain | Not listed the data on physical activity |
| Focht BC 2005 | Not evaluated physical activity or pain | Not listed the data on physical activity |
| Focht BC 2014 | Not evaluated physical activity or pain | Not listed the data on physical activity |
| Foley A 2003 | Not evaluated physical activity or pain | Not listed the data on physical activity |
| Foo CN 2020 | No data available on physical activity or pain | Results of physical activity are not described in detail. |
| Foroughi N 2011 | Not evaluated physical activity or pain | Not listed the data on physical activity |
| Fransen M 2001 | Not evaluated physical activity or pain | Not listed the data on physical activity |
| Frost H 2002 | Not evaluated physical activity or pain | Not listed the data on physical activity |
| Fukumoto Y 2014 | Not evaluated physical activity or pain | Not listed the data on physical activity |
| Fukumoto Y 2017 | Not a pure control group | Control group received exercise intervention. |
| Ganji R 2018 | Not evaluated physical activity or pain | Not listed the data on physical activity |
| Gomiero AB 2018 | Not evaluated physical activity or pain | Not listed the data on physical activity |
| Goonasegaran AR 2022 | Not a pure control group | Control group received exercise intervention. |
| Goślińska J 2020 | Not evaluated physical activity or pain | Not listed the data on physical activity |
| Gränicher P 2020 | No data available on physical activity or pain | Results of physical activity are not described in detail. |
| Gür H 2002 | Not evaluated physical activity or pain | Not listed the data on physical activity |
| Gurjalwar I 2021 | Not evaluated physical activity or pain | Not listed the data on physical activity |
| Gurudut P 2018 | Not evaluated physical activity or pain | Not listed the data on physical activity |
| Halbert J 2001 | Not evaluated physical activity or pain | Not listed the data on physical activity |
| Hale LA 2012 | Not evaluated physical activity or pain | Not listed the data on physical activity |
| Hall M 2017 | Not a pure control group | Control group received exercise intervention. |
| Hall M 2018 | Not evaluated physical activity or pain | Not listed the data on physical activity |
| Hammer NM 2016 | Not evaluated physical activity or pain | Not listed the data on physical activity |
| Harper SA 2019 | Not a pure control group | Control group received exercise intervention. |
| Harris-Hayes M 2020 | Not evaluated physical activity or pain | Not listed the data on physical activity |
| Hausmann LRM 2018 | Not evaluated physical activity or pain | Not listed the data on physical activity |
| Healey EL 2016 | Not evaluated physical activity or pain | Not listed the data on physical activity |
| Hejdysz K 2020 | Not evaluated physical activity or pain | Not listed the data on physical activity |
| Helminen EE 2015 | Not evaluated physical activity or pain | Not listed the data on physical activity |
| Henriksen M 2014 | Not evaluated physical activity or pain | Not listed the data on physical activity |
| Henriksen M 2016 | Not evaluated physical activity or pain | Not listed the data on physical activity |
| Herbold JA 2014 | Not evaluated physical activity or pain | Not listed the data on physical activity |
| Hermann A 2016 | No data available on physical activity or pain | No data available prior to total knee arthroplasty. |
| Hernandez D 2019 | Not a pure control group | Control group received exercise intervention. |
| Heuts PH 2005 | Not evaluated physical activity or pain | Not listed the data on physical activity |
| Hinman RS 2019 | Not a pure control group | Control group received educational intervention. |
| Hiyama Y 2012 | Not a pure control group | Control group received exercise intervention. |
| Hoeksma HL 2006 | Not evaluated physical activity or pain | Not listed the data on physical activity |
| Holm B 2012 | Several diseases included | Included post total knee arthroplasty patients. |
| Holm PM 2020 | Not evaluated physical activity or pain | Not listed the data on physical activity |
| Holsgaard-Larsen A 2018 | Not a pure control group | Control group received pharmacotherapy. |
| Hopman-Rock M 2000 | Not evaluated physical activity or pain | Not listed the data on physical activity |
| Hsu YI 2021 | Not evaluated physical activity or pain | Not listed the data on physical activity |
| Hu X 2020 | Not evaluated physical activity or pain | Not listed the data on physical activity |
| Huang MH 2003 | Not evaluated physical activity or pain | Not listed the data on physical activity |
| Huang SW 2012 | Not evaluated physical activity or pain | Not listed the data on physical activity |
| Huber EO 2013 | Not evaluated physical activity or pain | Not listed the data on physical activity |
| Huber EO 2015 | Not a pure control group | Control group received educational intervention. |
| Hunt MA 2013 | Not evaluated physical activity or pain | Not listed the data on physical activity |
| Hunt MA 2018 | Not evaluated physical activity or pain | Not listed the data on physical activity |
| Hurley DA 2020 | Several diseases included | Patients with low back pain are included |
| Huysmans E 2021 | Not evaluated physical activity or pain | Not listed the data on physical activity |
| Ikuta F 2020 | Not evaluated physical activity or pain | Not listed the data on physical activity |
| Isaramalai SA 2018 | Not evaluated physical activity or pain | Not listed the data on physical activity |
| Jansen MJ 2011 | Not evaluated physical activity or pain | Not listed the data on physical activity |
| Javed S 2021 | Not a pure control group | Control group received exercise intervention. |
| Jegu AG 2014 | Not evaluated physical activity or pain | Not listed the data on physical activity |
| Jepson P 2016 | No data available on physical activity or pain | No data available prior to total knee arthroplasty. |
| Jigami H 2012 | Not evaluated physical activity or pain | Not listed the data on physical activity |
| Jones A 2012 | Not a relevant intervention | Verification with and without the use of a cane |
| Jorge RT 2015 | Not evaluated physical activity or pain | Not listed the data on physical activity |
| Joshi S 2019 | Not a pure control group | Control group received exercise intervention. |
| JU SB 2015 | Not evaluated physical activity or pain | Not listed the data on physical activity |
| Kabiri S 2018 | Not evaluated physical activity or pain | Not listed the data on physical activity |
| Kang JW 2011 | Not evaluated physical activity or pain | Not listed the data on physical activity |
| Kao MJ 2012 | Not evaluated physical activity or pain | Not listed the data on physical activity |
| Karimi N 2021 | Not a pure control group | Control group received exercise intervention. |
| Karp JF 2019 | Not evaluated physical activity or pain | Not listed the data on physical activity |
| Kars Fertelli T 2019 | Not evaluated physical activity or pain | Not listed the data on physical activity |
| Keays SL 2016 | Not evaluated physical activity or pain | Not listed the data on physical activity |
| Keefe FJ 1990 | Not evaluated physical activity or pain | Not listed the data on physical activity |
| Keefe FJ 1996 | Not evaluated physical activity or pain | Not listed the data on physical activity |
| Keefe FJ 1999 | Not evaluated physical activity or pain | Not listed the data on physical activity |
| Keefe FJ 2004 | Not evaluated physical activity or pain | Not listed the data on physical activity |
| Keeley T 2015 | Not evaluated physical activity or pain | Not listed the data on physical activity |
| Keogh JW 2018 | Not evaluated physical activity or pain | Not listed the data on physical activity |
| Khachian A 2020 | Not evaluated physical activity or pain | Not listed the data on physical activity |
| Khruakhorn S 2021 | Not evaluated physical activity or pain | Not listed the data on physical activity |
| Kılıç F 2020 | Not evaluated physical activity or pain | Not listed the data on physical activity |
| Kim IS 2012 | Not evaluated physical activity or pain | Not listed the data on physical activity |
| Kloek CJJ 2018 | Not a pure control group | Control group received usual physical therapy according to the Dutch physical therapy guidelines. |
| Knoop J 2013 | Not evaluated physical activity or pain | Not listed the data on physical activity |
| Knoop J 2014 | Not evaluated physical activity or pain | Not listed the data on physical activity |
| Kotteeswaran K 2018 | Not a relevant intervention | Both group received therapeutic ultrasound |
| Kotteeswaran K 2021 | Not evaluated physical activity or pain | Not listed the data on physical activity |
| Kovar PA 1992 | Not evaluated physical activity or pain | Not listed the data on physical activity |
| Krasilshchikov O 2011 | Not evaluated physical activity or pain | Not listed the data on physical activity |
| Krauß I 2014 | Not evaluated physical activity or pain | Not listed the data on physical activity |
| Krauss I 2020 | Not evaluated physical activity or pain | Not listed the data on physical activity |
| Kulisch Á 2014 | Not evaluated physical activity or pain | Not listed the data on physical activity |
| Kumar S 2013 | Not evaluated physical activity or pain | Not listed the data on physical activity |
| Kuntz AB 2018 | Not evaluated physical activity or pain | Not listed the data on physical activity |
| Kuptniratsaikul V 2002 | Not evaluated physical activity or pain | Not listed the data on physical activity |
| Kuptniratsaikul V 2019 | Not a pure control group | Control group received home-based exercise. |
| Kuru Çolak T 2017 | Not a pure control group | Control group received exercise intervention. |
| Laforest S 2012 | Not evaluated physical activity or pain | Not listed the data on physical activity |
| Lai Z 2019 | Not evaluated physical activity or pain | Not listed the data on physical activity |
| Larose J 2013 | Not evaluated physical activity or pain | Not listed the data on pain |
| Lawford BJ 2018 | Not evaluated physical activity or pain | Not listed the data on physical activity |
| Leal-blanquet J 2013 | Not evaluated physical activity or pain | Not listed the data on physical activity |
| Ledingham A 2020 | Wrong study type | Non-randomized trial |
| Lee AC 2017 | Not a pure control group | Control group received exercise intervention. |
| Lee HJ 2009 | Not evaluated physical activity or pain | Not listed the data on physical activity |
| Legha A 2020 | Not evaluated physical activity or pain | Not listed the data on physical activity |
| Levinger P 2018 | Not evaluated physical activity or pain | Not listed the data on physical activity |
| Li K 2006 | Not a relevant intervention | Both group received multiple interventions, e.g. cold or thermal therapy, electrotherapy and aquatic therapy. |
| Liao CD 2015 | Several diseases included | Participants who underwent total knee arthroplasty |
| Lim BW 1984 | No data available on physical activity or pain | Results of physical activity are not described in detail. |
| Lim BW 2002 | Not a pure control group | Control group received exercise intervention. |
| Lin YT 2020 | Not evaluated physical activity or pain | Not listed the data on physical activity |
| Liu J 2019a | Not evaluated physical activity or pain | Not listed the data on physical activity |
| Liu J 2019b | Not evaluated physical activity or pain | Not listed the data on physical activity |
| Lluch E 2018 | Not evaluated physical activity or pain | Not listed the data on physical activity |
| Loew L 2017 | Not evaluated physical activity or pain | Not listed the data on physical activity |
| Losina E 2017 | Not evaluated physical activity or pain | Not listed the data on physical activity |
| Lü J 2017 | Not evaluated physical activity or pain | Not listed the data on physical activity |
| Lun V 2015 | Not evaluated physical activity or pain | Not listed the data on physical activity |
| Mallen CD 2017 | Not evaluated physical activity or pain | Not listed the data on physical activity |
| Mangani I 2006 | Not evaluated physical activity or pain | Not listed the data on physical activity |
| Manisha N 2015 | Not evaluated physical activity or pain | Not listed the data on physical activity |
| Marconcin P 2018 | Not evaluated physical activity or pain | Not listed the data on physical activity |
| Marra CA 2012 | No data available on physical activity or pain | Results of physical activity are not described in detail. |
| Mascarin NC 2012 | Not evaluated physical activity or pain | Not listed the data on physical activity |
| Mat Eil Ismail MS 2016 | No data available on physical activity or pain | No data available prior to total knee arthroplasty. |
| Matassi F 2014 | Not evaluated physical activity or pain | Not listed the data on physical activity |
| Mazzuca SA 2004 | Not evaluated physical activity or pain | Not listed the data on physical activity |
| McCaffrey R 2019 | Not a pure control group | Control group received exercise intervention. |
| McCarthy CJ 2004a | Not evaluated physical activity or pain | Not listed the data on physical activity |
| McCarthy CJ 2004b | Not evaluated physical activity or pain | Not listed the data on physical activity |
| McCarthy CJ 2005 | Not evaluated physical activity or pain | Not listed the data on physical activity |
| Mecklenburg G 2018 | Not evaluated physical activity or pain | Not listed the data on physical activity |
| Messier SP 2013 | Not evaluated physical activity or pain | Not listed the data on physical activity |
| Messier SP 2021 | Not evaluated physical activity or pain | Not listed the data on physical activity |
| Mikesky AE 2006 | Not evaluated physical activity or pain | Not listed the data on physical activity |
| Mitchell, C. 2005 | Not evaluated physical activity or pain | Not listed the data on physical activity |
| Moe RH 2016 | Not evaluated physical activity or pain | Not listed the data on physical activity |
| Moezy A 2021 | Not a pure control group | Control group received exercise intervention. |
| Moffet H 2015 | Several diseases included | Participants who underwent total knee arthroplasty |
| Moghadam EB 2017 | Not evaluated physical activity or pain | Not listed the data on physical activity |
| Moody J 2012 | Not evaluated physical activity or pain | Not listed the data on physical activity |
| Moonaz SH 2015 | Not evaluated physical activity or pain | Not listed the data on physical activity |
| Moreira VMPS 2021 | Not evaluated physical activity or pain | Not listed the data on physical activity |
| Moura-Fernandes MC 2020 | Not evaluated physical activity or pain | Not listed the data on physical activity |
| Multanen J 2017 | Not evaluated physical activity or pain | Not listed the data on physical activity |
| Munugoda IP 2020 | Not a relevant intervention | Surgical intervention were included |
| Munukka M 2016 | Not evaluated physical activity or pain | Not listed the data on physical activity |
| Munukka M 2020 | Wrong study type | Secondary analysis |
| Murphy SL 2012 | Not evaluated physical activity or pain | Not listed the data on pain |
| Narang S 2014 | Not evaluated physical activity or pain | Not listed the data on physical activity |
| Neelapala YVR 2018 | Not evaluated physical activity or pain | Not listed the data on physical activity |
| Nejati P 2015 | Not evaluated physical activity or pain | Not listed the data on physical activity |
| Nelligan RK 2021a | Not a pure control group | Control group received educational intervention. |
| Nelligan RK 2021b | Not a pure control group | Control group received educational intervention. |
| Nicklas BJ 2005 | Not evaluated physical activity or pain | Not listed the data on physical activity |
| Nunez M 2006 | Not evaluated physical activity or pain | Not listed the data on physical activity |
| Odole AC 2013 | Not evaluated physical activity or pain | Not listed the data on physical activity |
| Odole AC 2014 | Not evaluated physical activity or pain | Not listed the data on physical activity |
| Oh SL 2021 | Not a pure control group | Control group received educational intervention. |
| Ojoawo AO 2016 | Not evaluated physical activity or pain | Not listed the data on physical activity |
| Oladapo MO 2016 | Not evaluated physical activity or pain | Not listed the data on physical activity |
| Oliveira AM 2012 | Not evaluated physical activity or pain | Not listed the data on physical activity |
| Olsen AL 2022 | Not a pure control group | Control group received educational intervention. |
| O'moore KA 2018 | Not evaluated physical activity or pain | Not listed the data on physical activity |
| Onwunzo CN 2021 | Not evaluated physical activity or pain | Not listed the data on physical activity |
| Oosting E 2012 | Not a pure control group | Control group received educational intervention. |
| O'Reilly SC 1999 | Not evaluated physical activity or pain | Not listed the data on physical activity |
| Østerås H 2017 | Not evaluated physical activity or pain | Not listed the data on physical activity |
| Östlind E 2022 | Not a pure control group | Control group received educational intervention. |
| Ota S 2021 | Not a pure control group | Control group received exercise intervention. |
| Ozdincler AR 2005 | Not evaluated physical activity or pain | Not listed the data on physical activity |
| Pariser D 2005 | Not evaluated physical activity or pain | Not listed the data on physical activity |
| Park J 2014 | Not evaluated physical activity or pain | Not listed the data on physical activity |
| Park J 2016 | Not evaluated physical activity or pain | Not listed the data on physical activity |
| Park J 2017 | Not evaluated physical activity or pain | Not listed the data on physical activity |
| Park J 2020 | Not evaluated physical activity or pain | Not listed the data on physical activity |
| Pelle T 2020 | Not a pure control group | Both groups were allowed to visit physical therapist, which might be applied some interventions. |
| Péloquin L 1999 | Not evaluated physical activity or pain | Not listed the data on physical activity |
| Penninx BW 2001 | Not evaluated physical activity or pain | Not listed the data on physical activity |
| Penninx BW 2004 | Not evaluated physical activity or pain | Not listed the data on physical activity |
| Perez-Huerta BD 2020 | Not evaluated physical activity or pain | Not listed the data on physical activity |
| Peterson MG 1993 | Not evaluated physical activity or pain | Not listed the data on physical activity |
| Petrella RJ 2000 | Not a relevant intervention | Both group applied nonsteroidal medication. |
| Peungsuwan P 2014 | Not evaluated physical activity or pain | Not listed the data on physical activity |
| Pinto D 2013 | Not evaluated physical activity or pain | Not listed the data on physical activity |
| Pisters MF 2010 | Not evaluated physical activity or pain | Not listed the data on physical activity |
| Piyakhachornrot N 2011 | Not evaluated physical activity or pain | Not listed the data on physical activity |
| Posadzki P 2011 | Not evaluated physical activity or pain | Not listed the data on physical activity |
| Poulsen E 2013 | Not evaluated physical activity or pain | Not listed the data on physical activity |
| Praharsitha R 2019 | Not a pure control group | Control group received exercise intervention. |
| Pua YH 2011 | Not evaluated physical activity or pain | Not listed the data on physical activity |
| Quicke JG 2017 | Wrong study type | Secondary analysis |
| Quicke JG 2018 | Wrong study type | Secondary analysis |
| Quilty B 2003 | Not evaluated physical activity or pain | Not listed the data on physical activity |
| Raeissadat SA 2018 | Not evaluated physical activity or pain | Not listed the data on physical activity |
| Rafiq MT 2021 | Not evaluated physical activity or pain | Not listed the data on physical activity |
| Raj NB 2019 | Not evaluated physical activity or pain | Not listed the data on physical activity |
| Rashid SA 2019 | Not a pure control group | Control group received exercise intervention. |
| Ravaud P 2004 | Not evaluated physical activity or pain | Not listed the data on physical activity |
| Razek RA 2014 | Not evaluated physical activity or pain | Not listed the data on physical activity |
| Reid DA 2011 | Not evaluated physical activity or pain | Not listed the data on physical activity |
| Rezasoltani Z 2020 | Not evaluated physical activity or pain | Not listed the data on physical activity |
| Rezende M 2021 | Not evaluated physical activity or pain | Not listed the data on physical activity |
| Riddle DL 2019 | Not evaluated physical activity or pain | Not listed the data on physical activity |
| Rini C 2015 | Not evaluated physical activity or pain | Not listed the data on physical activity |
| Rogers MW 2011a | Not evaluated physical activity or pain | Not listed the data on physical activity |
| Rogers MW 2011b | Not evaluated physical activity or pain | Not listed the data on physical activity |
| Rogers MW 2012 | Not evaluated physical activity or pain | Not listed the data on physical activity |
| Rooks DS 2006 | Not evaluated physical activity or pain | Not listed the data on physical activity |
| Rosedale R 2014 | Not evaluated physical activity or pain | Not listed the data on physical activity |
| Saeed HH 2021 | Not a pure control group | Control group received exercise intervention. |
| Saffari M 2018 | Not evaluated physical activity or pain | Not listed the data on physical activity |
| Salacinski AJ 2012 | No data available on physical activity or pain | Results of physical activity are not described in detail. |
| Sameer AG 2021 | Not evaluated physical activity or pain | Not listed the data on physical activity |
| Samuel SD 2014 | Not evaluated physical activity or pain | Not listed the data on physical activity |
| Samut G 2015 | Not evaluated physical activity or pain | Not listed the data on physical activity |
| Saraboon Y 2015 | Not evaluated physical activity or pain | Not listed the data on physical activity |
| Saw MM 2016 | Not evaluated physical activity or pain | Not listed the data on physical activity |
| Sayers SP 2012 | Not evaluated physical activity or pain | Not listed the data on physical activity |
| Schepens SL 2012 | Not evaluated physical activity or pain | Not listed the data on physical activity |
| Schilke JM 1996 | Not evaluated physical activity or pain | Not listed the data on physical activity |
| Schoo AM 2005 | Not evaluated physical activity or pain | Not listed the data on physical activity |
| Sedaghatnezhad P 2021 | Not evaluated physical activity or pain | Not listed the data on physical activity |
| Segal NA 2015 | Not evaluated physical activity or pain | Not listed the data on physical activity |
| Sekir U 2005 | Not evaluated physical activity or pain | Not listed the data on physical activity |
| Shaffer KM 2020 | Not evaluated physical activity or pain | Not listed the data on physical activity |
| Sharma M 2018 | Not evaluated physical activity or pain | Not listed the data on physical activity |
| Sharma P 2014 | Not evaluated physical activity or pain | Not listed the data on physical activity |
| Sharma SS 2013 | Not evaluated physical activity or pain | Not listed the data on physical activity |
| Shellington EM 2019 | Not evaluated physical activity or pain | Not listed the data on physical activity |
| Simão AP 2012 | Not evaluated physical activity or pain | Not listed the data on physical activity |
| Singh A 2011 | Not evaluated physical activity or pain | Not listed the data on physical activity |
| Singh J 2011 | Not evaluated physical activity or pain | Not listed the data on physical activity |
| Singh S 2016 | Not evaluated physical activity or pain | Not listed the data on physical activity |
| Singh S 2017 | Not evaluated physical activity or pain | Not listed the data on physical activity |
| Skoffer B 2018 | Wrong study type | Secondary analysis |
| Skou ST 2015a | Not evaluated physical activity or pain | Not listed the data on physical activity |
| Skou ST 2015b | Not evaluated physical activity or pain | Not listed the data on physical activity |
| Skou ST 2016 | Not evaluated physical activity or pain | Not listed the data on physical activity |
| Skou ST 2018 | Not evaluated physical activity or pain | Not listed the data on physical activity |
| Skrepnik N 2017 | Not evaluated physical activity or pain | Not listed the data on physical activity |
| Slawson DC 2014 | Not evaluated physical activity or pain | Not listed the data on physical activity |
| Smith MT 2015 | Not evaluated physical activity or pain | Not listed the data on physical activity |
| Smith TO 2012 | Not evaluated physical activity or pain | Not listed the data on physical activity |
| Somers TJ 2012 | Not evaluated physical activity or pain | Not listed the data on physical activity |
| Song R 2003 | Not evaluated physical activity or pain | Not listed the data on physical activity |
| Sperber NR 2013 | Not evaluated physical activity or pain | Not listed the data on physical activity |
| Squiers M 2021 | Not evaluated physical activity or pain | Not listed the data on physical activity |
| Stanton TR 2020 | Not a pure control group | Control group received both exercise and educational intervention. |
| Steinhilber B 2017 | Not evaluated physical activity or pain | Not listed the data on physical activity |
| Sullivan T 1998 | Not a pure control group | Control group received educational intervention. |
| Suzuki Y 2019 | Not a pure control group | Control group received exercise intervention. |
| Svege I 2015 | Not a pure control group | Control group received educational intervention. |
| Svege I 2016 | Not evaluated physical activity or pain | Not listed the data on physical activity |
| Swank AM 2011 | Not evaluated physical activity or pain | Not listed the data on physical activity |
| Taglietti M 2018 | Not evaluated physical activity or pain | Not listed the data on physical activity |
| Tak E 2005 | Not evaluated physical activity or pain | Not listed the data on physical activity |
| Takacs J 2017 | Not evaluated physical activity or pain | Not listed the data on physical activity |
| Talbot LA 2003 | Not a pure control group | Control group received educational intervention. |
| Tamin TZ 2018 | Not evaluated physical activity or pain | Not listed the data on physical activity |
| Tariq K 2020 | Not evaluated physical activity or pain | Not listed the data on physical activity |
| Teirlinck CH 2016 | Not evaluated physical activity or pain | Not listed the data on physical activity |
| Teixeira PEP 2011 | Not evaluated physical activity or pain | Not listed the data on physical activity |
| Teja KR 2020 | Not a pure control group | Control group received exercise intervention. |
| Tejedor Varillas A 2012 | Not evaluated physical activity or pain | Not listed the data on physical activity |
| Thiengwittayaporn S 2021 | Not a pure control group | Control group received exercise intervention. |
| Thomas KS 2002 | Not evaluated physical activity or pain | Not listed the data on physical activity |
| Thompson AR 2020 | Not evaluated physical activity or pain | Not listed the data on physical activity |
| Thorstensson CA 2005 | Not evaluated physical activity or pain | Not listed the data on physical activity |
| Topp R 2002 | Not evaluated physical activity or pain | Not listed the data on physical activity |
| Topp R 2017 | Not evaluated physical activity or pain | Not listed the data on physical activity |
| Trudeau KJ 2015 | Not evaluated physical activity or pain | Not listed the data on physical activity |
| Tsai PF 2013 | Not evaluated physical activity or pain | Not listed the data on physical activity |
| Tüzün S 2012 | Not evaluated physical activity or pain | Not listed the data on physical activity |
| Uzunkulaoğlu A 2018 | Not a pure control group | Control group received exercise intervention. |
| Uzunkulaoğlu A 2020 | Not a pure control group | Control group received exercise intervention. |
| Vaghela N 2020 | Not evaluated physical activity or pain | Not listed the data on physical activity |
| van Baar ME 1998a | Not a pure control group | Control group received educational intervention and medication. |
| van Baar ME 1998b | Not evaluated physical activity or pain | Not listed the data on physical activity |
| van Baar ME 2001 | Not a pure control group | Control group received educational intervention. |
| van Ginckel A 2016 | Not evaluated physical activity or pain | Not listed the data on physical activity |
| Varah Y 2020 | Not evaluated physical activity or pain | Not listed the data on physical activity |
| Vassão PG 2020 | Not evaluated physical activity or pain | Not listed the data on physical activity |
| Veenhof C 2005 | No data available on physical activity or pain | Results of physical activity are not described in detail. |
| Veenhof C 2006 | Not a pure control group | Control group received exercise intervention. |
| Vina ER 2016 | Not evaluated physical activity or pain | Not listed the data on physical activity |
| Vincent KR 2019 | Not evaluated physical activity or pain | Not listed the data on physical activity |
| Vitiello MV 2013 | Not evaluated physical activity or pain | Not listed the data on physical activity |
| Von Korff M 2012 | No data available on physical activity or pain | Results of physical activity are not described in detail. |
| Wallis JA 2011 | Not evaluated physical activity or pain | Not listed the data on physical activity |
| Walsh N 2020 | Several diseases included | Patients with low back pain are included |
| Wang C 2009 | Not evaluated physical activity or pain | Not listed the data on physical activity |
| Wang C 2016 | Not a pure control group | Control group received exercise intervention. |
| Wang J 2020 | Not evaluated physical activity or pain | Not listed the data on physical activity |
| Wang L 2020 | Not evaluated physical activity or pain | Not listed the data on physical activity |
| Wang P 2016a | Not a pure control group | Control group received exercise intervention. |
| Wang P 2016b | Not evaluated physical activity or pain | Not listed the data on physical activity |
| Wang SY 2012 | Not evaluated physical activity or pain | Not listed the data on physical activity |
| Wang TJ 2007 | Not evaluated physical activity or pain | Not listed the data on pain |
| Wang TJ 2011 | Not evaluated physical activity or pain | Not listed the data on physical activity |
| Wang X 2020 | Not evaluated physical activity or pain | Not listed the data on physical activity |
| Williams NH 2011 | No data available on physical activity or pain | Results of pain are not described in detail. |
| Xiao C 2020 | Not evaluated physical activity or pain | Not listed the data on physical activity |
| Xiao Z 2021 | Not evaluated physical activity or pain | Not listed the data on physical activity |
| Yañez-Álvarez A 2020 | Not evaluated physical activity or pain | Not listed the data on physical activity |
| Ye J 2020a | Not evaluated physical activity or pain | Not listed the data on physical activity |
| Ye J 2020b | Not evaluated physical activity or pain | Not listed the data on physical activity |
| Yilmaz M 2019 | Not evaluated physical activity or pain | Not listed the data on physical activity |
| Yip YB 2007 | Not a pure control group | Control group received exercise intervention. |
| Zeng R 2015 | Not evaluated physical activity or pain | Not listed the data on physical activity |
| Zhang Zhiwang 2020 | Not evaluated physical activity or pain | Not listed the data on physical activity |
| Zhu Q 2016 | Not evaluated physical activity or pain | Not listed the data on physical activity |
| Zhu Q 2017 | Not evaluated physical activity or pain | Not listed the data on physical activity |
| Zietek P 2015 | Several diseases included | Participants who underwent total knee arthroplasty |
